# Defect-Limited Efficiency of Pnictogen Chalcohalide Solar Cells

**DOI:** 10.1021/acs.chemmater.5c03275

**Published:** 2026-02-20

**Authors:** Cibrán López, Seán R. Kavanagh, Pol Benítez, Edgardo Saucedo, Aron Walsh, David O. Scanlon, Claudio Cazorla

**Affiliations:** † Departament de Física, Universitat Politècnica de Catalunya, 08034 Barcelona, Spain; ‡ Barcelona Research Center in Multiscale Science and Engineering, Universitat Politècnica de Catalunya, 08019 Barcelona, Spain; § 98029 Harvard University Center for the Environment, Cambridge, Massachusetts 02138, United States; ∥ Departament d’Enginyeria Electrònica, Universitat Politècnica de Catalunya, 08034 Barcelona, Spain; ⊥ Thomas Young Centre and Department of Materials, 4615 Imperial College London, Exhibition Road, London SW7 2AZ, U.K.; # Department of Physics, Ewha Womans University, 52 Ewhayeodae-gil, Seodaemun-gu, Seoul 03760, South Korea; ¶ School of Chemistry, University of Birmingham, Birmingham B15 2TT, U.K.; ∇ Institució Catalana de Recerca i Estudis Avançats (ICREA), Passeig Lluís Companys 23, 08010 Barcelona, Spain

## Abstract

Pnictogen chalcohalides (MChX) have recently emerged as promising nontoxic and environmentally friendly photovoltaic absorbers, combining strong light absorption coefficients with favorable low-temperature synthesis conditions. Despite these advantages and reported optimized morphologies, device efficiencies remain below 10%, far from their ideal radiative limit. To uncover the origin of these performance losses, we present a systematic and fully consistent first-principles investigation of the defect chemistry across the Bi-based chalcohalide family. Our results reveal a complex defect landscape dominated by chalcogen vacancies of low formation energy, which act as deep nonradiative recombination centers. Despite their moderate charge-carrier capture coefficients, the high equilibrium concentrations of these defects reduce the theoretical maximum efficiencies by 6% in BiSeI and by 10% in BiSeBr. In contrast, sulfur vacancies in BiSI and BiSBr are comparatively benign, presenting smaller capture coefficients due to weaker electron–phonon coupling. Interestingly, despite its huge nonradiative charge-carrier recombination rate, BiSeI presents the best conversion efficiency among all four compounds owing to its most suitable bandgap for outdoor photovoltaic applications. Our findings identify defect chemistry as a critical bottleneck in MChX solar cells and propose chalcogen-rich synthesis conditions and targeted anion substitutions as effective strategies for mitigation of detrimental vacancies.

## Graphical abstract

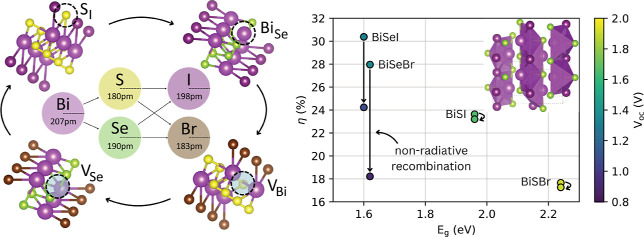

## 1 Introduction

The global transition toward sustainable and low-carbon energy systems has placed photovoltaics (PV) at the forefront of research and technological innovation.^1^ As a clean, renewable, and abundant energy source, PV provides a direct route to decarbonizing electricity generation. However, photovoltaic technologies must satisfy stringent performance criteria, including high power conversion efficiency (PCE), long-term stability, scalability, and low-cost manufacturing while maintaining environmental sustainability.^2^

Pnictogen chalcohalides (MChX, Figure 1a) have recently emerged as a promising class of photovoltaic absorbers owing to their nontoxicity, bandgaps in the range of 1.2–2.1 eV, light absorption coefficients spanning from 25 to 66 μm^–1^,^3–5^ exceptional thermodynamic stability,^6,7^ and low synthesis temperatures (below 300 °C).^7,8^ MChX materials are often described as “perovskite-inspired” semiconductors^9^ since their electronic structures resemble those of lead-halide perovskites, despite crystallizing in different crystal lattices. In particular, such materials are believed to present defect tolerance, stemming from the antibonding and bonding character of their valence and conduction bands, respectively. Actually, the closely related family of binary chalcogenides (e.g., Sb_2_S_3_, Sb_2_Se_3_, and Bi_2_S_3_) has been shown to exhibit genuine defect-tolerance features.^10–12^

**Figure 1:**
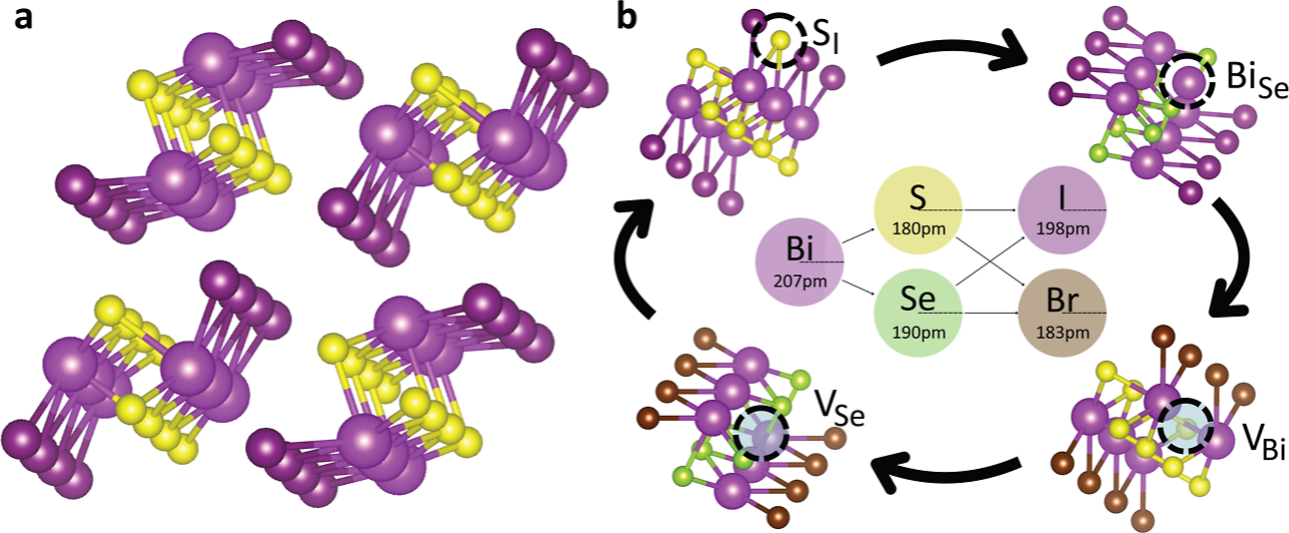
MChX photovoltaic absorbers. (a) MChX orthorhombic crystal structure (space group *Pnma*) characterized by columnar motifs held together by weak dispersion forces. Pnictogen, chalcogen, and halide atoms are represented by violet, yellow, and dark pink spheres, respectively. (b) A systematic study of MChX point defects disentangles general trends. Four different Bi-based chalcohalides and all possible intrinsic point defects have been systematically analyzed in this study. The van der Waals radius of each species is marked for reference.

Despite these encouraging prospects, experimental PCEs of MChX solar cells remain below 10%, far from their ideal Shockley–Queisser limit obtained from detailed balance (e.g., 30% in BiSeI).^13,14^ Understanding the origin of this performance decline is therefore essential for realizing their full technological potential and enabling large-scale deployment. While efficiency losses can arise from synthesis routes, morphology, or even device architecture, recent studies indicate that such extrinsic factors are not the primary bottlenecks in MChX photovoltaics.^5,9^ Instead, intrinsic crystalline defects are increasingly recognized as the decisive factor.

Extended, high-dimensional defects such as grain boundaries, dislocations, and precipitates, although typically detrimental in many semiconductors, are found to be noncritical in MChX^8^ since their quasi-one-dimensional chain-like framework appears to hinder charge recombination.^15–17^ On the other hand, point defects, often overlooked in early assessments, may dominate nonradiative recombination and ultimately constrain device performance.^18,19^

Recent theoretical studies on BiSeI^19^ and their binary counterparts Sb_2_S_3_ ^20^ and Sb_2_Se_3_ ^21^ have identified chalcogen vacancies as dominant recombination centers, drastically reducing their maximum PCE. Experimental works have also reported similar defect behavior in related materials.^22^ Consequently, sulfur and selenium vacancies (*V*_S_ and *V*_Se_) might play a significant detrimental role in the broader MChX family. Yet, their relative impact on device performance may differ substantially depending on the interplay of the local chalcogen–halogen environment, which motivates a systematic, cross-material computational investigation of point defects on pnictogen chalcohalides.

Systematically estimating point-defect behavior (e.g., formation energies and carrier capture coefficients) in MChX is highly nontrivial.^23,24^ First-principles calculations of defect formation energies and carrier capture processes^25^ are computationally demanding and highly sensitive to methodological choices.^26^ Comparisons across different studies are often complicated^27–29^ by variations in input parameters or approximations, often relying on error cancellation,^30^ which hinders finding solid and reproducible trends. By contrast, a consistent, family-wide study performed with identical computational protocols offers a robust framework to disentangle chemical effects from methodological artifacts (Figure 1b).^19,31^

Here, we present a comprehensive investigation of the defect chemistry of four representative pnictogen chalcohalides (BiSI, BiSeI, BiSBr, and BiSeBr) using advanced first-principles density functional theory (DFT) calculations. A systematic comparison of all of them allows identification of selenium vacancies as the most detrimental defects within this family; in particular, they introduce deep electronic transition states near the Fermi level and enhance electron–phonon coupling, thus promoting nonradiative recombination in BiSeI and BiSeBr. In contrast, sulfur vacancies in BiSI and BiSBr are comparatively benign owing to weaker electron–phonon coupling, despite presenting similar formation energies. This striking contrast originates from the larger ionic radius and higher polarizability of selenium atoms, which enhance lattice distortions around *V*_Se_, suggesting that the chemical environment (specifically, the interplay between chalcogen and halogen anions) dictates charge recombination activity in MChX. This work establishes design principles for defect engineering in pnictogen chalcohalides while also offering a generalizable framework applicable to other emergent photovoltaic materials.

## 2 Results

MChX crystallize into an orthorhombic phase (space group *Pnma*) characterized by one-dimensional columns held together by weak van der Waals forces (Figure 1a),^9^ closely resembling the structure of binary pnictogen chalcogenides (Sb_2_S_3_, Sb_2_Se_3_, and Bi_2_Se_3_).^15^ Our DFT geometry optimizations for MChX yield lattice parameters and electronic structures that are in very good agreement with the available experimental data^3,9^ (Supporting Information and Table S1). According to previous first-principles calculations and experimental observations,^7,8^ MChX are thermodynamically stable against phase separation into secondary phases (Figure S1) and present indirect bandgaps in the range of 1 to 2 eV (Table S1 and Figure S2).

### 2.1 Defect Formation Energies

Point defects play a pivotal role in the optoelectronic performance of semiconductors. They can compensate for doping-induced electrical imbalance, shift the Fermi energy level (*E*_F_), and act as deep nonradiative charge recombination centers that quench carrier lifetimes and limit photovoltaic performance. To assess their impact on MChX, we computed the formation energies (*E*_f_) of all relevant point defects as a function of the Fermi energy level (delimited by the valence band maximum and conduction band minimum, VBM ≤ *E*_F_ ≤ CBM) for BiSI, BiSeI, BiSBr, and BiSeBr. In practice, low (high) defect formation energies imply a high (low) equilibrium concentration of defects, as follows from the corresponding Boltzmann-like distribution.

Defect formation energy diagrams (Figures S3–S11) provide a compact map of thermodynamic stability: the slope of each line reflects the defect charge state, the intersections mark charge–state transition levels (acceptance or donation of electrons), and the relative position of the curves identifies which defects are most favorable under given synthesis conditions. In terms of PV performance, the most detrimental defects are those with low formation energies near the self-consistent Fermi level, *E*_F_^sc^.^32,33^ This equilibrium Fermi level ensures charge neutrality across the defect and carrier populations in the system,^34^ and it can be shifted with temperature and growth conditions.

The influence of the chemical environment on *E*_f_ enters explicitly through the atomic chemical potentials (Methods), which bound the accessible growth window during synthesis. For MChX, the two pnictogen-poor and chalcogen-poor limits are particularly relevant (Table 1). These limits capture the experimentally relevant conditions where defect chemistry is expected to differ most strongly, while intermediate states interpolate between them. During experimental synthesis, chalcogen-poor conditions are frequently encountered owing to the high volatility of Se and S atoms at elevated temperatures.^7,8^

**I tbl1:** Calculated Chemical Potential Limits for the Thermodynamic Stability Window of MChX Compounds^a^

	Bi-poor			S/Se-poor		
material	μ_Bi_	μ_S/Se_	μ_I/Br_	μ_Bi_	μ_S/Se_	μ_I/Br_
BiSI	–0.87	0	–0.46	0	–0.58	–0.75
BiSeI	–0.97	0	–0.42	0	–0.65	–0.75
BiSBr	–0.90	0	–0.67	0	–0.60	–0.97
BiSeBr	–1.03	0	–0.63	0	–0.68	–0.97

a Bi-poor and S/Se-poor conditions are the two limiting cases. Chemical potentials are expressed in units of eV.

In the following sections, we focus on vacancies and antisites since these point defects consistently emerge as the lowest-energy across MChX. In fact, according to our calculations, interstitials exhibit significantly higher formation energies^19^ (Figure S3), in agreement with experimental observations in binary chalcogenides,^35–38^ and are therefore excluded from our next detailed analysis.

#### 2.1.1 BiSI

At room temperature, *E*_F_^sc^ lies 1.57 eV above the VBM in Bi-poor environments and 1.70 eV in S-poor environments, considering defect populations set at a realistic annealing temperature of 550 K (Figure S4). The calculated *E*_F_^sc^ exhibits a weak dependence on temperature (Figure S5), which denotes robust n-type character.

BiSI exhibits a high density of deep midgap charge-transition states that are likely to act as recombination centers (Figure 2a). These mostly include antisites, with Bi_S_ showing three transitions (+5/+3, +3/+1, and +1/–1) at 1.06–1.23 eV from the VBM, complemented by Bi_I_ (+5/0) and *S*_Bi_ (+1/–1) at 1.14 and 1.07 eV, respectively. Sulfur vacancies with a charge-transition state (+2/0) represent deep levels, positioned at 1.23 eV from the VBM.

**Figure 2:**
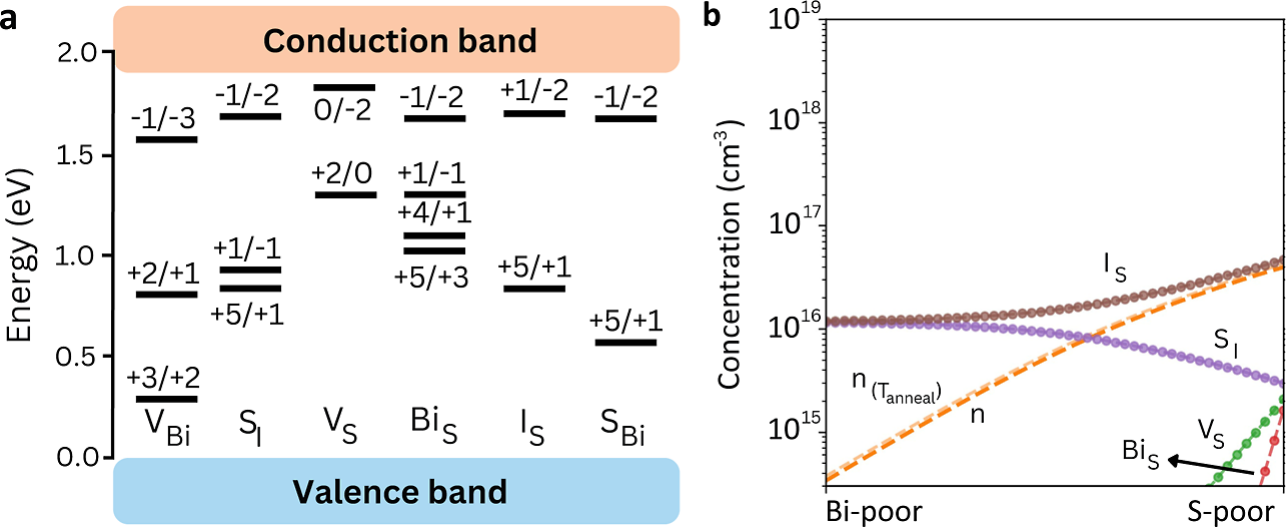
Point-defect chemistry in BiSI. (a) Charge-state transition levels of the most prominent (i.e., lowest energy) point defects determined for BiSI. (b) Defect concentrations of BiSI considering an annealing temperature of 550 K. The chemical stability region is delimited by S-poor (μ_Bi_, μ_S_, and μ_I_) = (0, −0.58, and −0.75) eV and Bi-poor conditions (μ_Bi_, μ_S_, and μ_I_) = (−0.87, 0, and −0.46) eV.

Under S-poor conditions, *V*_S_ exhibits a formation energy of 0.77 eV at the self-consistent Fermi level, *E*_F_^sc^ (Figure S5). Several antisite defects present also very low formation energies at *E*_F_^sc^, the most critical cases being *S*_I_ (0.51 eV) and Bi_S_ (0.54 eV). Bi-poor synthesis conditions generally result in higher formation energies. For example, the formation energy of *V*_S_ increases to 1.34 eV. However, certain antisite defects still exhibit very low *E*_f_: 0.35 eV for *S*_I_ and 0.80 eV for *S*_Bi_.

These results have a direct effect on the equilibrium defect populations, which are highest under S-poor conditions with, for example, 4.7 × 10^16^ cm^–3^ for *I*_S_, 3.0 × 10^15^ cm^–3^ for *S*_I_, 2.1 × 10^15^ cm^–3^ for *V*_S_, and 1.6 × 10^15^ cm^–3^ for Bi_S_ (Figure 2b). On the contrary, these populations are moderate under Bi-poor conditions with, for example, 1.2 × 10^16^ cm^–3^ for *I*_S_ and *S*_I_.

#### 2.1.2 BiSeI

In BiSeI, the defect landscape is similar to that of BiSI but with slightly different energetics (Figure S6). *V*_Se_ forms at 0.82 eV under Se-poor and 1.47 eV under Bi-poor conditions. Bi_Se_ is highly favorable, requiring only 0.34 eV under Se-poor growth but increasing to 2.04 eV in Bi-poor environments. The equilibrium Fermi level is again pinned high in the gap, 1.38 eV above the VBM under Bi-poor, and 1.46 eV under Se-poor conditions, with little dependence on annealing temperature (Figure S7).

Similarly to BiSI, BiSeI exhibits deep levels such as Bi_Se_ (+5/+1) positioned at 1.10 eV, Bi_I_ (+4/0) at 1.12 eV from the VBM, Se_I_ (+1/–1) at 0.87 eV, Se_Bi_ (+1/–1) at 1.06 eV, and *V*_Se_ (+2/0) at 1.08 eV (Figure 3a). While in BiSeI there are fewer deep charge-transition states than in BiSI, the parallels between the two iodides indicate a shared tendency for potentially recombination-active centers.

**Figure 3:**
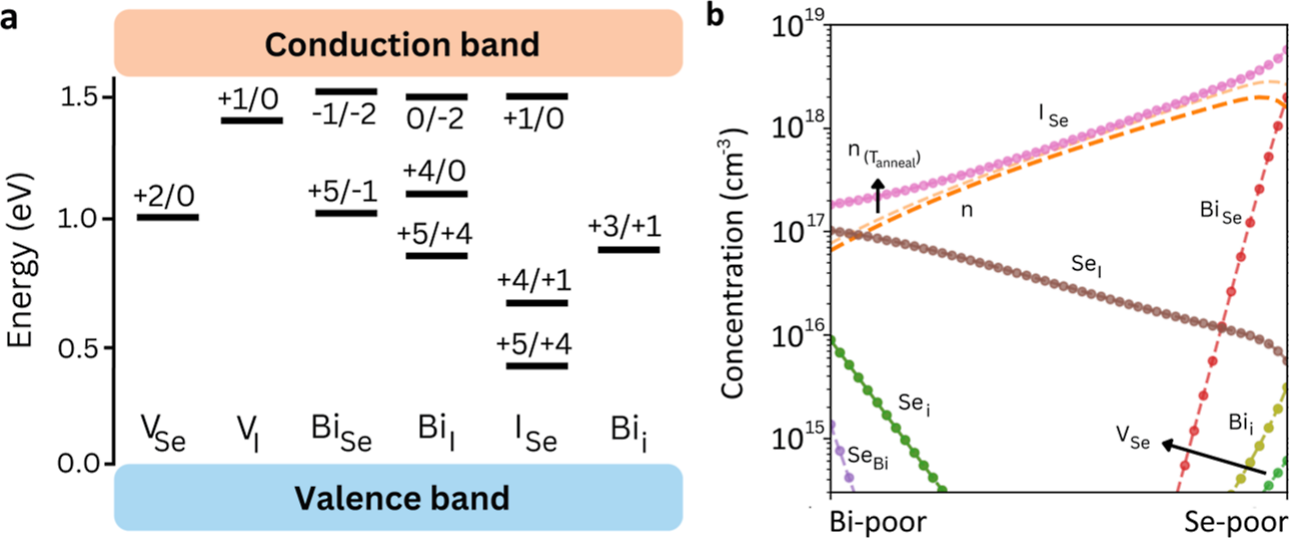
Point-defect chemistry in BiSeI. (a). Charge-state transition levels of the most prominent (i.e., lowest energy) point defects determined for BiSeI. (b). Defect concentrations of BiSeI considering an annealing temperature of 550 K. The chemical stability region is delimited by Se-poor (μ_Bi_, μ_Se_, and μ_I_) = (0, −0.65, and −0.75) eV and Bi-poor conditions (μ_Bi_, μ_Se_, and μ_I_) = (−0.97, 0, and −0.42) eV.

The most critical antisites (Figure S6) are Bi_Se_ (0.34 eV) and *I*_Se_ (0.43 eV) under Se-poor conditions, while under Bi-poor conditions are *I*_Se_ (0.68 eV) and Se_I_ (0.38 eV). As in BiSI, the formation energies of vacancies are slightly higher than those of antisites. However, *V*_Se_ emerges as a key recombination-active defect with *E*_f_ = 0.82 eV, this formation energy being slightly lower than that of *V*_S_ in BiSI.

Once again, we found that the equilibrium defect populations are highest under Se-poor conditions, with 5.8 × 10^18^ cm^–3^ for *I*_Se_, 5.6 × 10^15^ cm^–3^ for Se_I_, 6.1 × 10^14^ cm^–3^ for *V*_Se_, and 2.0 × 10^18^ cm^–3^ for Bi_Se_ (Figure 3b). Meanwhile, under Bi-poor conditions, we obtain 1.8 × 10^17^ cm^–3^ for *I*_Se_ and 1.0 × 10^17^ cm^–3^ for Se_I_. These equilibrium defect concentrations, in general, are higher than those found in BiSI. Consequently, it can be inferred that in BiSeI, trap-mediated recombination would probably be more pronounced.

#### 2.1.3 BiSBr

BiSBr (Figure S8) shows the strongest n-type tendency since *E*_F_^sc^ is pinned at 1.82 eV above the VBM under Bi-poor and shifts further to 1.98 eV in S-poor conditions (Figure S9). Deep levels arise from both cationic and anionic sites (Figure 4a): Bi_S_ (+3/–1) at 1.34 eV from the VBM, Bi_Br_ (+4/+2, +2/0) at 1.25 and 1.47 eV, respectively, *S*_Bi_ (+1/–1) at 1.35 eV, *V*_S_ (+2/0) at 1.50 eV, and *V*_Bi_ (+1/–1) at 1.38 eV. These states reduce the dominance of antisite deep transitions while introducing an additional recombination pathway through the Bi vacancy.

**Figure 4:**
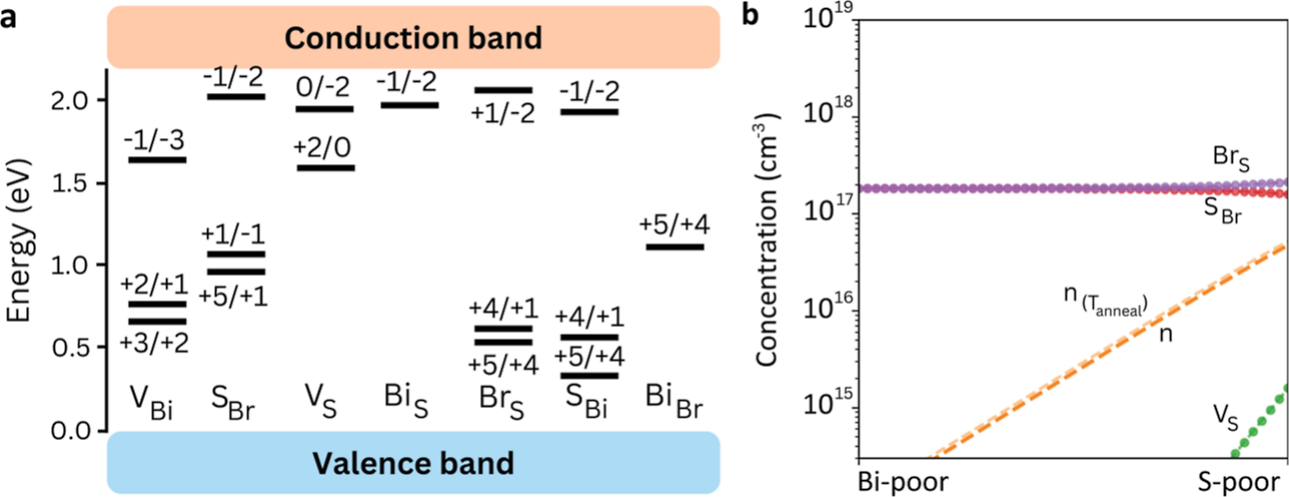
Point-defect chemistry in BiSBr. (a) Charge-state transition levels of the most prominent (i.e., lowest energy) point defects determined for BiSBr. (b) Defect concentrations of BiSBr considering an annealing temperature of 550 K. The chemical stability region is delimited by S-poor (μ_Bi_, μ_S_, and μ_Br_) = (0, −0.60, and −0.97) eV and Bi-poor conditions (μ_Bi_, μ_S_, and μ_Br_) = (−0.90, 0, and −0.67) eV.

In BiSBr, the lowest-energy antisites are *S*_Br_ and Br_S_, with *E*_f_ = 0.34 and 0.72 eV under S-poor growth, respectively. Under Bi-poor conditions, *S*_Br_ becomes particularly easy to form, with *E*_f_ dropping to 0.20 eV, while *S*_Bi_ also remains competitive (0.73 eV). Although vacancy formation is energetically less favorable, *V*_S_ again emerges as the lowest-energy case, with *E*_f_ = 0.79 eV under S-poor, closely resembling previous cases. This hierarchy reflects the structural penalty for removing atoms from the quasi-1D columns, in contrast to antisites, which require minimal rearrangement.

The equilibrium defect populations were found to closely resemble those in BiSI, with 2.1 × 10^17^ cm^–3^ for Br_S_, 1.6 × 10^17^ cm^–3^ for *S*_Br_, and 1.6 × 10^15^ cm^–3^ for *V*_S_ in S-poor conditions and 1.8 × 10^17^ cm^–3^ for Br_S_ and *S*_Br_ in Bi-poor conditions (Figure 4b). As a result, it would be expected for BiSBr to have trap-mediated efficiency losses similar to those of BiSI.

#### 2.1.4 BiSeBr

BiSeBr displays the richest set of midgap transitions (Figure 5a), including Bi_Se_ (+5/–1) at 0.94 eV from the VBM, Bi_Br_ (+4/+2 and +2/0) at 0.76 and 1.14 eV, respectively, Br_Bi_ (0/–2) at 0.82 eV, Se_Bi_ (+1/–1) at 1.10 eV, *V*_Se_ (+2/0) at 1.08 eV, and *V*_Bi_ (+1/–1) at 0.99 eV. This diversity exceeds that of BiSBr and the iodides, marking BiSeBr as the most defect-sensitive compound in the series.

**Figure 5:**
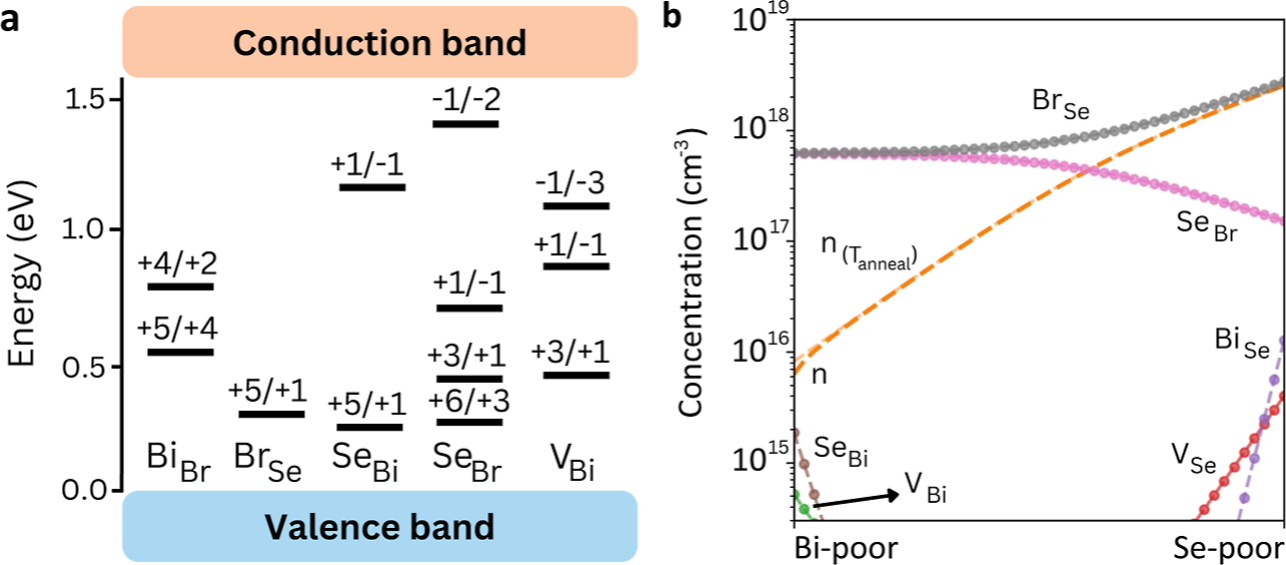
Point-defect chemistry in BiSeBr. (a). Charge-state transition levels of the most prominent (i.e., lowest energy) point defects determined for BiSeBr. (b). Defect concentrations of BiSeBr considering an annealing temperature of 550 K. The chemical stability region is delimited by Se-poor (μ_Bi_, μ_Se_, and μ_Br_) = (0, −0.68, and −0.97) eV and Bi-poor conditions (μ_Bi_, μ_Se_, and μ_Br_) = (−1.03, 0, and −0.63) eV.

In BiSeBr, Se-related defects dominate (Figure S10). *V*_Se_ forms at 0.74 eV under Se-poor conditions and 1.42 eV under Bi-poor. Bi_Se_ is also competitive under Se-poor conditions, with *E*_f_ = 0.56 eV, but not so under Bi-poor growth since its formation energy then increases to 2.43 eV. The equilibrium Fermi level lies 1.33 eV above the VBM under Bi-poor conditions and 1.49 eV in Se-poor conditions, indicating once again a robust n-type character (Figure S11).

The most critical antisites in Se-poor environments are Se_Br_ (0.44 eV) and Br_Se_ (0.49 eV) (Figure S10). Under Bi-poor conditions, Se_Br_ (0.26 eV) and Se_Bi_ (0.59 eV) remain highly favorable. Vacancies, though less favorable than antisites, still yield relatively low formation energies with *V*_Se_ (0.74 eV) acting as a likely nonradiative recombination center, quite close in energy to *V*_S_ in BiSI.

Again, the equilibrium defect populations are elevated under Se-poor conditions, with 2.8 × 10^18^ cm^–3^ for Br_Se_, 1.5 × 10^17^ cm^–3^ for Se_Br_, 1.3 × 10^16^ cm^–3^ for Se_Br_, and 4.0 × 10^15^ cm^–3^ for *V*_Se_ (Figure 5b). Under Bi-poor conditions, however, these equilibrium concentrations noticeably decrease: 6.2 × 10^17^ cm^–3^ for Br_Se_ and Se_Br_, 1.9 × 10^15^ cm^–3^ for Se_Bi_, and 5.2 × 10^14^ cm^–3^ for *V*_Bi_. These equilibrium defect concentrations are similar to those found in BiSeI and slightly higher than for BiSBr; thus, it can be expected that BiSeBr, along with BiSeI, presents more trap-mediated recombination than BiSI and BiSBr.

#### 2.1.5 General Trends

Clear and systematic trends emerge from the defect formation results obtained for BiSI, BiSeI, BiSBr, and BiSeBr (Figures 2–5). Our calculations show that *E*_F_^sc^ depends only weakly on annealing, indicating a robust n-type character across the MChX family. This behavior aligns with experimental observations that p-type doping is extremely challenging in these materials.^8^ Under chalcogen-poor conditions, Bi_Ch_ antisites consistently rank among the lowest energy defects. This preference originates from the similar atomic environments found around Bi and chalcogen positions along the quasi-one-dimensional chains (Figure 1a). Likewise, chalcogen-on-halogen substitutions (Ch_X_) repeatedly show very low formation energies, as seen for both S- and Se-based compounds, which can be rationalized by the comparable ionic radii of these species (Figure 1b). While the formation of vacancies is in general energetically less favorable, *V*_Ch_ consistently emerges as the dominant vacancy type. In a Bi-poor environment, the formation of Ch_X_ antisites remains the most favorable across all four materials, systematically followed by chalcogen-on-pnictogen (Ch_M_) and halogen-on-chalcogen (X_Ch_) substitutions. At the same time, *V*_M_ becomes the vacancy species with the lowest formation energies.

These general defect formation energy trends can be intuitively understood in terms of the quasi-one-dimensional crystal structure characteristic of MChX. Point defects are largely confined to the atomic columns in which they form, minimizing interactions with neighboring chains. Vacancies, which require substantial lattice relaxation within a column, exhibit moderate formation energies, whereas interstitials are even more energetically costly due to the extensive lattice disruption they introduce. By contrast, antisite defects, involving the replacement of one atom by another within the same column, require minimal structural rearrangement and are therefore easier to form. These patterns are consistent with prior observations in columnar pnictogen chalcogenides such as Sb_2_S_3_ and Sb_2_Se_3_.^18,39^

### 2.2 Charge-Carrier Capture Coefficients

Carrier capture coefficients (*C*_*n*/*p*_) provide a quantitative measure of the strength of electron–phonon coupling during defect-mediated charge exchange. While defect formation energies determine which defects are most abundant, carrier capture coefficients define which of those defects are electronically harmful by dictating how efficiently they can trap and recombine carriers.^40^ Defects with high *C*_*n*/*p*_ values act as efficient nonradiative recombination centers, drastically reducing carrier lifetimes and photovoltaic performance. Within multiphonon emission theory,^23^ these coefficients are calculated from the overlap of vibrational wave functions corresponding to different charge states (Methods), whose equilibrium geometries are connected through a generalized configuration coordinate, *Q* (Figure 6).

**Figure 6:**
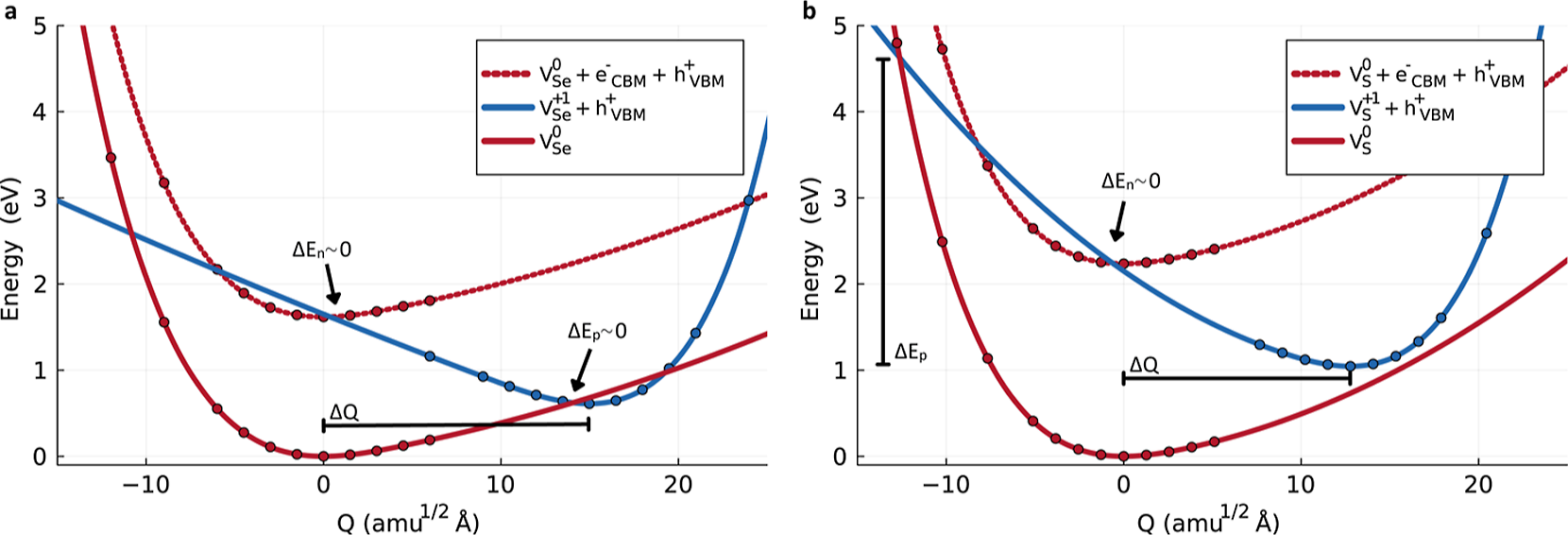
Configuration coordinate diagrams for BiSeBr and BiSBr. Configuration coordinate diagrams for (a) BiSeBr *V*_Se_ (0/+1) and (b) BiSBr *V*_S_ (0/+1). The dots represent potential energies computed from first-principles, and the solid lines are their corresponding quadratic spline interpolation and extrapolation. Δ*Q* represents the generalized distance between charge states and Δ*E*_*x*_ the electron (*n*)/hole (*p*) energy carrier capture barriers.

For BiSI (Figures S12, S13), the most prominent recombination-active centers are associated with sulfur-related defects (Table S2). We find particularly strong electron capture for the sulfur vacancy, with *C*_*n*_(*V*_S_, 0/+ 1) = 2.94 × 10^–8^ cm^3^/s, accompanied by a notable contribution from the antisite transition *C*_*n*_(Bi_S_, −1/0) = 2.40 × 10^–10^ cm^3^/s and *C*_*n*_(Bi_S_, +4/+ 5) = 1.06 × 10^–8^ cm^3^/s.

In BiSeI (Figures S14, S15), the selenium vacancy emerges as the dominant nonradiative defect (Table S3), exhibiting multiple strong capture channels: *C*_p_(*V*_Se_, 0/+ 1) = 5.89 × 10^–8^ cm^3^/s, *C*_*n*_(*V*_Se_, −1/0) = 6.71 × 10^–9^ cm^3^/s, and *C*_*n*_(*V*_Se_, +1/+ 2) = 1.24 × 10^–9^ cm^3^/s. These values are approximately 1 order of magnitude higher than those observed in BiSI, indicating a more severe role of chalcogen vacancies in the selenides. Additional contributions arise from antisite defects such as Bi_Se_, with transitions (−2/–1) and (−1/0) yielding coefficients of 5.53 × 10^–10^ cm^3^/s and 5.88 × 10^–10^ cm^3^/s, respectively. These antisites, while less active than *V*_Se_, still underscore the vulnerability of iodide compounds to midgap recombination centers.

In BiSBr (Figures S16, S17), the sulfur vacancy again proves decisive (Table S4), with *C*_*n*_(*V*_S_, −2/–1) = 4.66 × 10^–9^ cm^3^/s. Interestingly, this material also displays significant antisite activity, with *C*_*n*_(Bi_S_, +1/+ 2) = 1.69 × 10^–9^ cm^3^/s and *C*_*p*_(Bi_S_, +3/+ 4) = 5.99 × 10^–10^ cm^3^/s. Compared to the iodides, BiSBr distributes its recombination activity more evenly between vacancies and antisites, although it presents fewer recombination pathways.

BiSeBr (Figures S18, S19) follows the same overall pattern as BiSeI, with selenium vacancies acting as the most harmful recombination centers (Table S5). A very interesting result is the extraordinarily high hole capture rate *C*_*p*_(*V*_Se_, 0/+ 1) = 2.95 × 10^–5^ cm^3^/s, 3 orders of magnitude larger than in any other member of the series, firmly establishing *V*_Se_ as the dominant nonradiative defect in this compound. Additional strong recombination channels in BiSeBr include *C*_*n*_(*V*_Se_, −2/–1) = 1.31 × 10^–9^ cm^3^/s and the antisite transition *C*_*p*_(Bi_Se_, −1/0) = 8.85 × 10^–9^ cm^3^/s.

The origin of the unusually large hole capture coefficient *C*_*p*_ in BiSeBr is the almost zero hole energy capture barrier, Δ*E*_*p*_ (Figure 6a, Table S5), which strongly promotes hole trapping. In fact, the relationship between the calculated potential energy surfaces and the resulting carrier-capture coefficients can be understood through the “classical” energy capture barriers.^23,41^ These barriers are defined as the energy difference between the equilibrium geometry of the initial charge state and the crossing point of the corresponding potential energy surfaces (*q* → *q* + 1 for Δ*E*_*p*_ and *q* + 1 → *q* for Δ*E*_*n*_). In this framework, small barriers lead to fast carrier capture, whereas large barriers result in reduced capture coefficients. For example, BiSBr exhibits a much higher hole energy capture barrier than BiSeBr (Δ*E*_*p*_ = 3.58 eV, Figure 6b and Table S4) resulting in a *C*_*p*_ that is 8 orders of magnitude smaller. Nevertheless, both compounds display comparable electron energy capture barriers, Δ*E*_*n*_, leading to similarly moderate electron capture coefficients.

These results reveal that chalcogen vacancies (particularly *V*_Se_) dominate the nonradiative recombination landscape in MChX, with capture coefficients orders of magnitude larger than those of antisites. These values are closely in line with results obtained for binary chalcogenides, which amount to *C*_*n*_(*V*_S_, +1/+ 2) = 7.64 × 10^–6^ cm^3^/s and *C*_*p*_(*V*_S_, +1/+ 2) = 2.25 × 10^–8^ cm^3^/s for Sb_2_S_3_ ^20^ and *C*_*n*_(*V*_Se_, +1/+ 2) = 5.63 × 10^–6^ cm^3^/s and *C*_*p*_(*V*_Se_, +1/+ 2) = 1.22 × 10^–8^ cm^3^/s for Sb_2_Se_3_.^21^ For MChX, the sulfides exhibit electron–phonon coupling somewhat weaker than that of the selenides, explaining their lower recombination activity despite similar defect energetics. Antisites such as Bi_Ch_, although often abundant, generally display smaller capture coefficients and consequently can be regarded as comparatively benign.

### 2.3 Power Conversion Efficiencies

The previous analysis of defect formation energies and charge-carrier capture coefficients highlights the potential impact of intrinsic defects on nonradiative recombination in MChX absorbers. To place these effects in context, we first consider the ideal case: the detailed balance model,^13^ which neglects nonradiative carrier losses and establishes the theoretical radiative limits for the MChX family (Table 2 and Figure 7).

**II tbl2:** Estimated Power Conversion Efficiencies and Related Properties Considering Different Limits and Growth Conditions^a^

					nonradiative limit					
		radiative limit			Bi-poor			S/Se-poor		
material	*E*_g_ (eV)	η (%)	*V*_oc_ (V)	FF (%)	η (%)	*V*_oc_ (V)	FF (%)	η (%)	*V*_oc_ (V)	FF (%)
BiSI	1.96	23.63	1.66	92.08	23.63	1.66	92.08	23.20	1.66	90.66
BiSeI	1.60	30.38	1.33	90.54	30.38	1.33	90.54	24.23	1.08	88.81
BiSBr	2.24	17.65	1.92	92.94	17.65	1.92	92.94	17.26	1.91	91.25
BiSeBr	1.62	27.96	1.35	90.66	27.96	1.35	90.66	18.21	0.91	87.23

a Bandgap, power conversion efficiencies (η), open-circuit voltages (*V*_oc_), and FF for BiSI, BiSeI, BiSBr, and BiSeBr estimated at the radiative and nonradiative limits.

**Figure 7:**
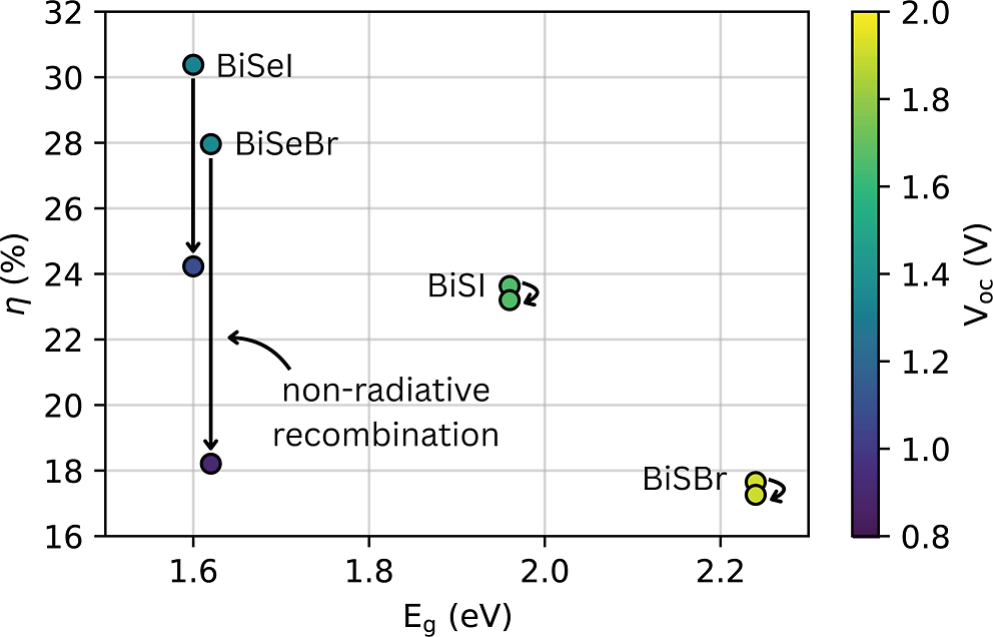
Impact of nonradiative recombination in the estimated power conversion efficiencies and open-circuit voltage. PCE and open-circuit voltage of BiSI, BiSeI, BiSBr, and BiSeBr as a function of bandgap. Colored points indicate the open-circuit voltage for each material with a heatmap scale. Arrows indicate the drop in efficiency from the radiative to the nonradiative limit under chalcogen-poor conditions.

At room temperature and accounting for thickness-dependent absorptivity in a 700 nm absorber layer, BiSI achieves a maximum PCE (η) of 23.63%, with an open-circuit voltage (*V*_oc_) of 1.66 V and a fill factor (FF) of 92.08%. BiSeI, benefiting from its narrower bandgap (Table II), reaches an ideal η of 30.38% with *V*_oc_ = 1.33 V and FF = 90.54%, underscoring the intrinsic suitability of the iodides as outdoor photovoltaic absorbers. Among the bromides, BiSBr exhibits the lowest predicted efficiency, with a detailed balance η of 17.65%, despite a comparatively high *V*_oc_ of 1.92 V and FF of 92.94%. Its poor efficiency arises directly from its wide bandgap (Table II), which severely limits photocurrent generation. BiSeBr, in contrast, performs on par with BiSeI, achieving a maximum η of 27.96% (*V*_oc_ = 1.35 V and FF = 90.66%). Taken together, these results demonstrate that S-based compounds inherently yield lower η values than Se-based compounds due to wider bandgaps of the former.

Despite these favorable radiative limits, experimental η values for MChX solar cells have not surpassed thus far 10%.^8,42,43^ While extrinsic factors such as film morphology, interface quality, and device architecture undoubtedly contribute, our results indicate that defect-assisted nonradiative recombination is also a critical bottleneck. To quantify its impact, we evaluated the defect-limited maximum η using the calculated carrier capture coefficients (*C*_*n*/*p*_), defect concentrations, and related parameters (Methods).^44^

Under Ch-poor synthesis conditions and an annealing temperature of 550 K, η decreases only marginally for the sulfides to 23.20% for BiSI and 17.26% for BiSBr, corresponding to a reduction of just 0.4% compared to the radiative limit (Table II). In contrast, for the selenides, the effect is much stronger: η drops to 24.23% for BiSeI and 18.21% for BiSeBr, representing losses of 6% and 10%, respectively (Figure 7). These reductions are accompanied by significant drops in *V*_oc_, falling to 1.08 V for BiSeI and 0.91 V for BiSeBr, while remaining nearly unchanged for BiSI and BiSBr. The FF is uniformly reduced by about 2% across all of the Bi-based chalcohalides. By comparison, Bi-poor conditions have a negligible influence, effectively minimizing the impact of trap-mediated recombination on photovoltaic performance (Table II).

Separate η calculations (Table S6), in which we isolate the recombination effects of chalcogen vacancies and Bi_Ch_ antisites, reveal that the efficiency loss arises entirely from chalcogen vacancies, which act as strong nonradiative centers. Antisite defects, despite their low formation energies, exhibit extremely small carrier capture coefficients and can therefore be regarded as electronically benign. A similar picture holds across the broader family: in BiSI and BiSBr, sulfur-related defects (*V*_S_, Bi_S_) produce negligible recombination losses, keeping their efficiencies effectively identical with the radiative limit. Thus, the dominant factor limiting nonradiative performance in MChX is the presence of chalcogen-site vacancies, whereas antisite defects appear comparatively harmless for device operation.

## 3 Discussion

The nonradiative efficiency losses in MChX (Table II and Figure 7) are comparable to those observed in other light absorber materials, such as Cu_2_ZnSnS_4_,^45^ Cu_2_ZnSnSe_4_,^40^ and CdTe.^31^ Both BiSeI and BiSeBr exhibit pronounced reductions in PCE relative to their radiative limits of approximately 6% and 10%, respectively. These losses are accompanied by marked decreases in open-circuit voltage (*V*_oc_ = 1.08 V for BiSeI and 0.91 V for BiSeBr) and FF (88.81% and 87.23%, respectively), reflecting a deterioration in electronic quality under illumination and a reduced charge-transport efficiency. Interestingly, this loss mechanism arises not from exceptionally strong recombination activity but from the high equilibrium concentration of selenium vacancies (*V*_Se_), whose relatively low formation energies (≈0.8 eV) lead to abundant deep-level traps. Hence, performance limitations in MChX absorbers are mainly driven by the prevalence of intrinsic point defects rather than by their individual recombination strengths. Effective passivation of these defects is, therefore, a promising avenue for improving device performance. Our first-principles results demonstrate that adopting Bi-poor synthesis conditions can significantly raise defect formation energies, thereby reducing their population. Under such conditions, the calculated efficiencies for BiSeI and BiSeBr are 30.38% and 27.96%, respectively, closely approaching their ideal detailed-balance limits.

A complementary route to mitigate these defect-induced PCE losses may involve targeted ion substitution, which provides a chemically guided approach to engineering defect tolerance. The similar van der Waals radii of Bi (*r*_Bi_ = 207 pm), Se (*r*_Se_ = 190 pm), and halogens such as Br (*r*_Br_ = 185 pm) or I (*r*_I_ = 198 pm) enable facile atomic interchange during synthesis, promoting antisite and vacancy formation. To counteract this atomic interchangeability, we propose controlled substitutions involving ions with larger size mismatches to hinder defect diffusion and reduce antisite formation while maintaining desirable optoelectronic characteristics. This rationale explains the enhanced defect tolerance observed in the sulfides BiSI and BiSBr, where the substantial ionic radius contrast among Bi, S (*r*_S_ = 180 pm), and halogens suppresses in part defect formation. These insights suggest that careful compositional tuning, particularly through I-on-Br substitution, offers a promising strategy to reduce the formation of defects in MChX semiconductors and boost their efficiency.

The findings presented in this study are significant for the field of photovoltaics, as they provide a pathway to enhance the efficiency of MChX light absorbers, which is a promising class of nontoxic and thermodynamically stable materials. By addressing defect chemistry and proposing effective passivation strategies, this study not only bridges the gap between theoretical predictions and experimental performance but also establishes a framework for designing more efficient solar absorbers with improved defect tolerance. These insights could pave the way for next-generation photovoltaic technologies with higher efficiencies and broader applicability.

Based on our theoretical findings, we suggest two promising experimental MChX research directions: (i) exploring ion substitution strategies (either full replacement or partial extrinsic doping) to suppress defect formation and weaken electron–phonon interactions that drive nonradiative recombination and (ii) pursuing synthesis under chalcogen-rich conditions, which would intrinsically minimize the concentration of detrimental vacancies and thereby reduce defect-mediated efficiency losses.

## 4 Conclusions

This work identifies intrinsic point defects as a key bottleneck limiting the photovoltaic performance of pnictogen chalcohalides. Through a systematic first-principles computational study involving BiSI, BiSeI, BiSBr, and BiSeBr, we show that chalcogen vacancies dominate nonradiative recombination, with selenium vacancies being particularly harmful due to their deep electronic transition states and strong electron–phonon coupling. In contrast, sulfur vacancies couple more weakly and are comparatively benign, highlighting the decisive influence of the local chemical environment on defect tolerance.

Our defect-limited efficiency analysis shows that while sulfides retain efficiencies close to their radiative limits, the selenides suffer losses of up to 10% on efficiency, accompanied by severe reductions in the open-circuit voltage. Crucially, we also demonstrate that synthesis conditions and targeted anion substitutions can mitigate the impact of harmful vacancies: Bi-poor growth suppresses *V*_Se_ formation, while S-for-Se or I-for-Br substitutions improve defect tolerance without sacrificing favorable optoelectronic properties. These findings establish general design principles for defect passivation in emergent optoelectronic materials, paving the way toward more efficient and defect-tolerant photovoltaic technologies.

## 5 Methods

### 5.1 First-Principles Calculations

Ab initio calculations based on DFT were performed to analyze point defects in MChX. These calculations were conducted with the VASP software package^46^ using the generalized gradient approximation to the exchange–correlation energy due to Perdew et al. (PBE).^47^ Since MChX are van der Waals materials, long-range dispersion interactions were taken into account through the D3 scheme.^48^ Spin–orbit coupling effects, which are particularly relevant for Bi-based MChX^4,19,32^ (Supporting Discussion), were taken into account along with range-separated hybrid functionals containing an exact Hartree–Fock exchange fraction of 25% (i.e., HSE + SOC^49–51^ ). All defective atomic structures were fully optimized at the HSE + D3+SOC level, a methodology shown to accurately reproduce experimental results for MChX and other similar materials.^3,19,52^ The projector augmented-wave method was used to represent the ionic cores,^53^ and for each element, the maximum possible number of valence electronic states was considered. Wave functions were represented in a plane-wave basis typically truncated at 300 eV. By using these parameters and a dense Γ-centered k-point grid for reciprocal space Brillouin zone integration of 2 × 1 × 2, the resulting energies were converged to within 1 meV per formula unit. In the geometry relaxations, a tolerance of 0.5 meV·Å^–1^ was imposed on the atomic forces. Defect calculations were performed in 3 × 2 × 1 (12.6 × 17.2 × 10.3 Å for BiSI, 12.8 × 18.1 × 11.3 Å for BiSeI, 12.1 × 16.1 × 9.5 Å for BiSBr, and 12.2 × 16.3 × 10.1 Å for BiSeBr) supercells.

### 5.2 Exploration of the Potential Energy Surface

Conventional approaches to generating defect configurations^33^ from pristine cells fail to find many lowering-energy conformations, which might have a crucial effect in the conclusions. Therefore, once a defect is generated (e.g., extraction of a bismuth atom), we look for distortions of the initial lattice configuration to locally explore the potential energy surface. These distortions were generated with the ShakeNBreak software package.^26,54^

Initially, all of the trial defect configurations were relaxed using Γ-point reciprocal space sampling and the HSE + D3 functional. Only the minimum-energy configurations were kept. Next, the ionic relaxations were repeated considering a larger k-point grid of 2 × 1 × 2 (Γ-centered). After that, the relaxations were repeated, considering SOC corrections. Finally, single-point energy calculations were performed using the previously converged electronic wave functions and equilibrium structures. Nonspherical contributions to the gradient of the density within the PAW spheres were taken into account to improve numerical accuracy.

### 5.3 Point-Defect Formation Energies

Point defects include vacancies, where an atom is removed from the lattice (e.g., *V*_Bi_), antisites, where an atom is replaced by another of a different species (e.g., *I*_S_), and interstitials, where an atom occupies a nonequilibrium lattice site (e.g., Br_i_). Computational approaches for studying these are well-established, relying on accurate first-principles energy calculations combined with exhaustive exploration of the defect local environment.^54–56^ For this work, we employed the supercell approach, which involves modeling point defects within sufficiently large supercells to minimize spurious interactions. We systematically analyzed all possible vacancy and antisite defects considering both neutral and charged states. The doped simulation package^57^ was used to generate defect structures and calculation inputs, determine chemical potential limits, and analyze the defects simulation results. The ShakeNBreak^54,58^ defect structure-search approach was employed, revealing numerous significant energy-lowering reconstructions, consistent with observations in similar low-dimensional chalcogenide systems.^20,39,59^

The formation energy of a point defect with charge *q*, *D* ^*q*^, can be expressed as^55^

$$\begin{array}{l} E_{f}\left(D^{q}\right)=E_{T}\left(D^{q}\right)-E_{T}\left(pristine\right)+\\ +qE_{F}-\underset{i}{\sum }n_{i}\mu_{i}+E_{corr}\left(D^{q}\right), \end{array}$$ 1

where *E*_*T*_(*D* ^*q*^) and *E*_*T*_(pristine) are the static energies of defected and pristine supercells (energy per formula unit), respectively, μ_*i*_ corresponds to chemical potential of species *i* (this is the energy required to extract one single atom), *n*_*i*_ the number of extracted atoms (positive or negative if extracted or added to the pristine cell, respectively), *E*_F_ the Fermi energy (energy needed to extract an electron), and *E*_corr_ the finite-size corrections based on spurious interactions between charged defects due to the periodic boundary conditions. The latter anisotropic correction scheme does not account for nonelectrostatic strain bias, which typically are assessed by performing supercell size scaling (and often are negligible within ∼10 Å simulation cells). Numerical tests were conducted on these finite-size effects, showing convergence of our defect formation energies to within 0.1 eV (Table S8). Moreover, defect state overlap dispersion was ruled out by analyzing total energies and checking for spurious delocalization and mixing across supercells.

Chemical potentials for substances consisting of elements *i* with concentrations *x*_*i*_ were computed via^60^

2 $$\sum_{i}x_{i}\left(\mu_{i}-\mu_{i}^{0}\right)=\Delta G_{f}^{0}\left(T\right)$$

with Δ*G*_f_^0^(*T*) representing the standard Gibbs formation free energy per atom and where all reference chemical potentials μ_*i*_^0^ are set to zero for elements in their standard states.

Here, we considered two different contributions to the finite-size energy correction: point-charge (due to the spurious electrostatic interactions of a defect with its images) and band-alignment (charged defects spuriously change the electrostatic potential of the system) corrections. Both corrections are computed together from an extension of the Freysoldt–Neugebauer–Van de Walle^55^ correction scheme to anisotropic materials,^56^ as implemented in the doped defect simulation package.^57^

### 5.4 Defect-Limited Efficiency

The strength of nonradiative recombination was quantified through the electron and hole capture coefficients corresponding to each charge state of the defect, as obtained with the CarrierCapture.jl package.^23,61^

In this theoretical framework, the maximum photovoltaic efficiency limited by defects^44^ for an absorber of thickness *W*, under an incident photon flux Φ at a photon energy *E* and bias voltage *V*, is expressed as

3 $$\eta =\max_{V}\left(\frac{JV}{q\int_{0}^{\infty }E\Phi \left(E\right)dE}\right)$$

where *q* denotes the elementary charge and *J* the maximum defect-limited current density, defined by

4 $$J\left(W,V\right)=J_{SC}\left(W\right)+J_{0}^{rad}\left(W,V\right)+J_{0}^{nonrad}\left(W,V\right)$$

Here, *J*_SC_ represents the short-circuit current, while *J*_0_^rad^ corresponds to the radiative saturation current. These two contributions describe recombination through photon emission, whereas *J*_0_^nonrad^ accounts for nonradiative processes

5 $$J_{SC}\left(W\right)=q\int_{0}^{\infty }a\left(E,W\right)\Phi \left(E\right)dE$$

6 $$\begin{array}{l} J_{0}^{rad}\left(W,V\right)=q\frac{2\pi }{c^{2}h^{3}}\left(1-e^{qV/k_{B}T}\right)\times \\ \times \int_{0}^{\infty }a\left(E,W\right){\left(e^{E/k_{B}T}-1\right)}^{-1}E^{2}dE \end{array}$$

7 $$J_{0}^{nonrad}\left(W,V\right)=-qWR^{SRH}\left(V\right)$$

with *a* the absorptivity (assuming unit quantum efficiency such that each absorbed photon produces an electron–hole pair). The Shockley–Read–Hall recombination rate is approximated as

8 $$R_{SRH}\approx \sum \Delta n/pN_{T}C_{n/p}$$

where Δ*n* and Δ*p* are the excess carrier concentrations, *N*_*T*_ the defect concentration, and the summation runs over all recombination-active centers. The capture coefficient,^23,40,62^ *C*_*n*/*p*_, depends on the electron–phonon interaction strength (*W*_ct_) and the overlap of vibrational wave functions (⟨ζ_*cm*_|Δ*Q*|ζ_*tn*_⟩)

9 $$\begin{array}{l} C_{n/p}=\Omega g\frac{2\pi }{\hbar }|W_{ct}{|}^{2}\sum_{m,n}w_{m}{\left\langle \zeta_{cm}\left|\Delta Q\right|\zeta_{tn}\right\rangle }^{2}\times \\ \times \delta \left(\Delta E_{n/p}+\epsilon_{cm}-\epsilon_{tn}\right), \end{array}$$

where Ω is the supercell volume, *g* the defect degeneracy, ζ the phonon wave function, and Δ*Q* the effective configuration coordinate, with *c* and *t* labeling free-carrier and trap states, respectively. The temperature dependence arises from the thermal occupation probability, *w*_*m*_ of the initial vibrational mode. To incorporate the role of capture dynamics on photovoltaic efficiency, calculations were carried out with the TLC code.^40^

The electron–phonon coupling matrix elements were computed as^23,61,62^

10 $$W_{ct}=\left(\epsilon_{t}-\epsilon_{c}\right)\frac{d\langle \zeta_{c}\left(0\right)|\zeta_{t}\left(Q\right)>}{dQ}$$

where *c*, *t* correspond to the perturbed band-edge state and the localized defect state, respectively, and the overlap between wave functions is explicitly calculated with DFT.

## Supplementary Material



## Data Availability

The data that support the findings of this study have been made publicly available,^63^ comprising the single-point energy and local potential calculations for all relaxed defects, along with all input files needed for reproducibility using the doped Python package.^57^

## Notes

DFT computational details of geometry optimization, thermodynamic stability, and defect formation energy calculations for MChX; convex-hull surfaces; energy-momentum bands structures; formation energies of interstitial point defects; formation energies of point defects in BiSI; temperature dependence of the Fermi level in BiSI; formation energies of point defects in BiSeI; temperature dependence of the Fermi level in BiSeI; formation energies of point defects in BiSBr; temperature dependence of the Fermi level in BiSBr; formation energies of point defects in BiSeBr; temperature dependence of the Fermi level in BiSeBr; configuration coordinate diagrams for *V*_S_ in BiSI; configuration coordinate diagrams for Bi_S_ in BiSI; configuration coordinate diagrams for *V*_Se_ in BiSeI; configuration coordinate diagrams for Bi_Se_ in BiSeI; configuration coordinate diagrams for *V*_S_ in BiSBr; configuration coordinate diagrams for Bi_S_ in BiSBr; configuration coordinate diagrams for *V*_Se_ in BiSeBr; configuration coordinate diagrams for Bi_Se_ in BiSeBr; DFT estimated and experimental optoelectronic and structural properties; calculated BiSI capture coefficients for each charge-carrier process involving *V*_S_ and Bi_S_; calculated BiSeI capture coefficients for each charge-carrier process involving *V*_Se_ and Bi_Se_; calculated BiSBr capture coefficients for each charge-carrier process involving *V*_S_ and Bi_S_; calculated BiSeBr capture coefficients for each charge-carrier process involving *V*_Se_ and Bi_Se_; separate contribution of chalcogen vacancies and Bi_Ch_ antisites to photovoltaic performance; photovoltaic performance of MChX in the radiative limit (at room temperature for a 700 nm absorber layer); size effects test on the formation energy of *V*_Se_ for BiSeBr; and impact of the employed DFT exchange–correlation functional on the obtained results and agreement with the experiments (PDF)
