# Supplementary material for: Defect-Limited Efficiency of Pnictogen Chalcohalide Solar Cells
Source: Chem Mater. 2026 Feb 20;38(6):2893–903. doi: 10.1021/acs.chemmater.5c03275 (PMC13019623; doi:10.1021/acs.chemmater.5c03275)
Supplement: Supplementary file 1 [file cm5c03275_si_001.pdf]

Supporting Information for "Defect–Limited Efficiency of Pnictogen Chalcohalide Solar Cells"

Cibrán López,[1,2] Seán R. Kavanagh,[3] Pol Benítez,[1,2] Edgardo Saucedo,[2,4]
Aron Walsh,[5,6] David O. Scanlon,[7] and Claudio Cazorla[1,2,8,*]

[1] *Departament de Física, Universitat Politècnica de Catalunya, 08034 Barcelona, Spain*
[2] *Barcelona Research Center in Multiscale Science and Engineering, Universitat Politècnica de Catalunya, 08019 Barcelona, Spain*
[3] *Harvard University Center for the Environment, Cambridge, Massachusetts 02138, United States*
[4] *Departament d'Enginyeria Electrònica, Universitat Politècnica de Catalunya, 08034 Barcelona, Spain*
[5] *Thomas Young Centre and Department of Materials, Imperial College London, Exhibition Road, London SW7 2AZ, UK*
[6] *Department of Physics, Ewha Womans University, 52 Ewhayeodae-gil, Seodaemun-gu, Seoul 03760, South Korea*
[7] *School of Chemistry, University of Birmingham, Birmingham B15 2TT, UK*
[8] *Institució Catalana de Recerca i Estudis Avançats (ICREA), Passeig Lluís Companys 23, 08010 Barcelona, Spain*

[*] claudio.cazorla@upc.edu

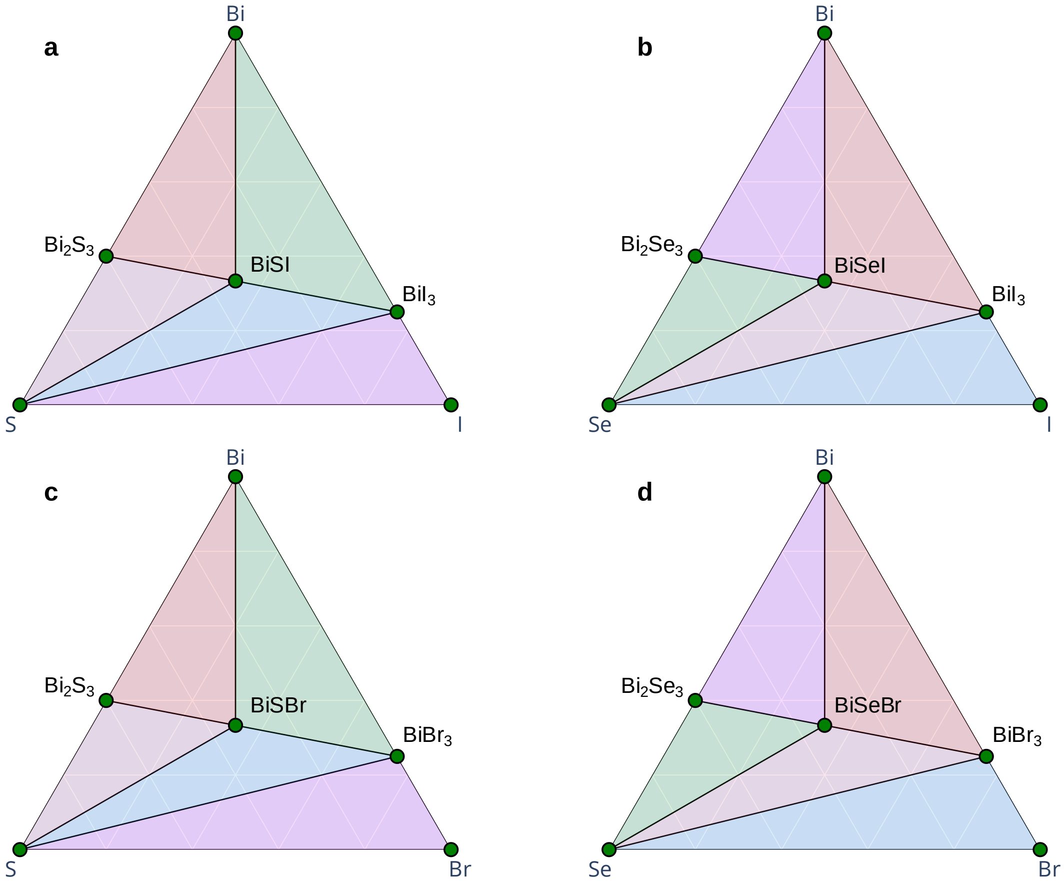

Fig. S1. Convex-hull surface of **a.** BiSI, **b.** BiSeI, **c.** BiSBr, and **d.** BiSeBr calculated with DFT methods. All of them are predicted to be thermodynamically stable against segregation into secondary phases because its formation enthalpy is negative relative to the convex-hull surface (all of them below -0.1 eV/atom).

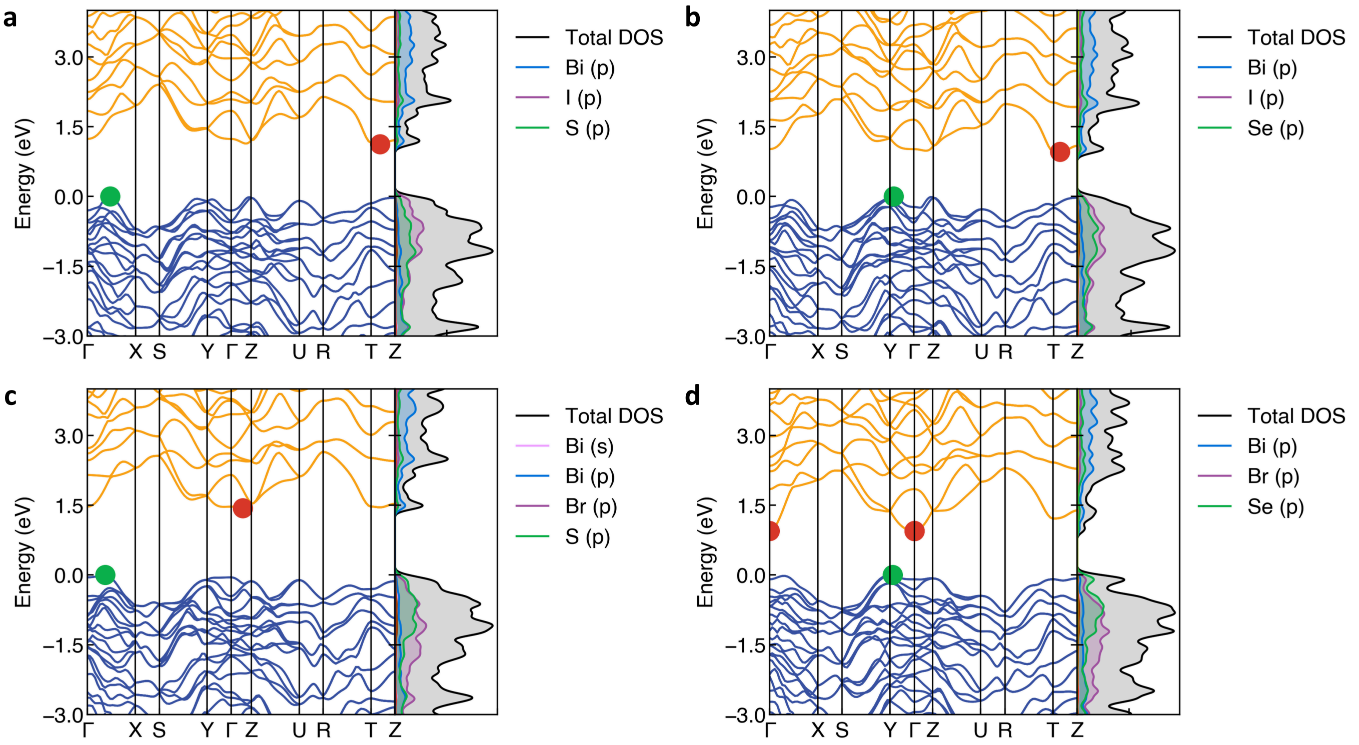

Fig. S2. Energy-momentum bands structures along high-symmetry **k**-paths in the Brillouin zone, computed at PBE_{sol}+SOC level of theory for **a.** BiSI, **b.** BiSeI, **c.** BiSBr, and **d.** BiSeBr. The figures were generated with the `sumo` toolkit [1].

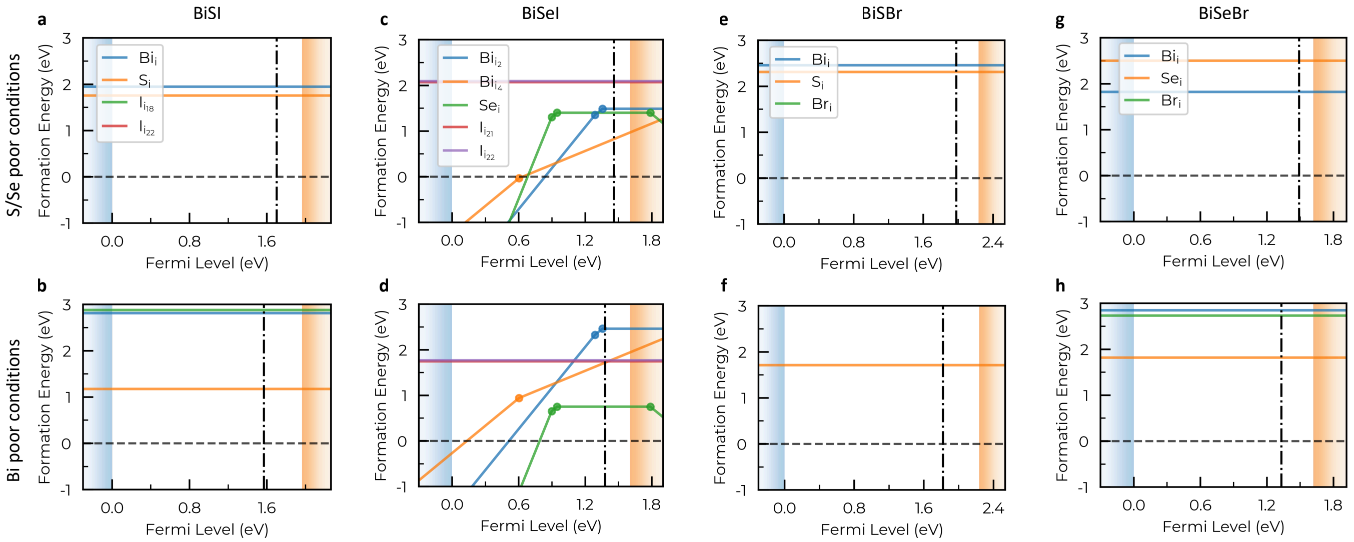

Fig. S3. Formation energies of interstitial point defects in MChX. Intestitial point defects for **a,b.** BiSI, **c,d.** BiSeI, **e,f.** BiSBr, and **g,h.** BiSeBr exihibit significantly high formation energies. The self-consistent Fermi level, E_F^{sc}, is represented with vertical dash-dot lines. Blue and orange shaded regions represent the valence and conduction bands, respectively.

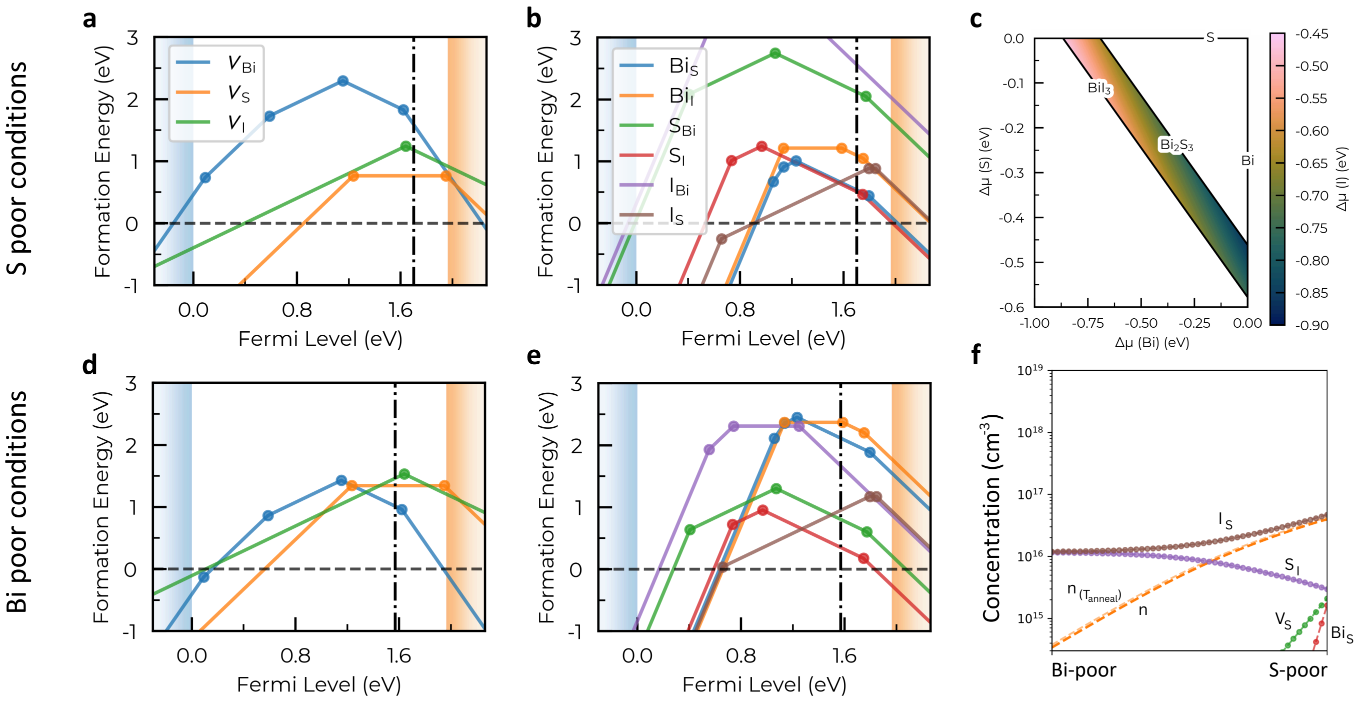

Fig. S4. Formation energies of point defects in BiSI. **a,b.** S-poor growth conditions. **d,e.** Bi-poor growth conditions. The self-consistent Fermi level, E_F^{sc}, is represented with vertical dash-dot lines, lying at 1.70 and 1.57 eV above the VBM (blue shaded region) for S-poor and Bi-poor growth conditions, respectively (the CBM is represented by the orange shaded region). **c.** Chemical stability region, delimited by S-poor (μ_{Bi}, μ_S, μ_I) = (0, -0.58, -0.75) eV and Bi-poor conditions (μ_{Bi}, μ_S, μ_I) = (-0.87, 0, -0.46) eV. **f.** Defect concentrations of BiSI considering an annealing temperature of 550 K.

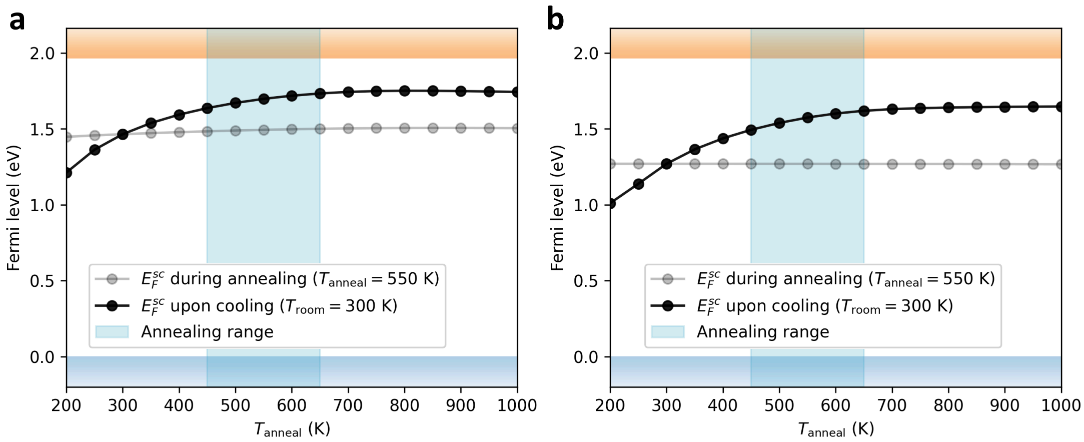

Fig. S5. Temperature dependence for BiSI of the Fermi level during annealing synthesis conditions for **a.** S-poor and **b.** Bi-poor conditions.

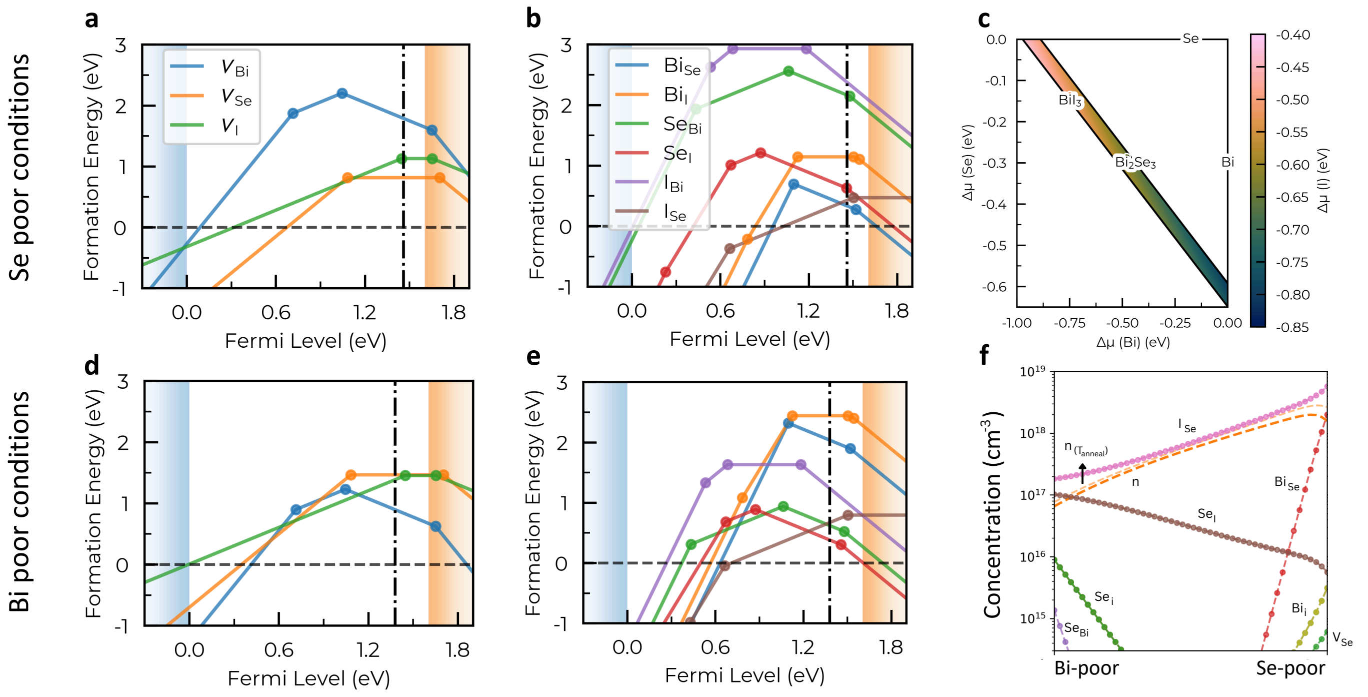

Fig. S6. Formation energies of point defects in BiSeI. **a,b.** S-poor growth conditions. **d,e.** Bi-poor growth conditions. The self-consistent Fermi level, E_F^{sc}, is represented with vertical dash-dot lines, lying at 1.46 and 1.38 eV above the VBM (blue shaded region) for Se-poor and Bi-poor growth conditions, respectively (the CBM is represented by the orange shaded region). **c.** Chemical stability region, delimited by Se-poor (μ_{Bi}, μ_{Se}, μ_I) = (0, -0.65, -0.75) eV and Bi-poor conditions (μ_{Bi}, μ_{Se}, μ_I) = (-0.97, 0, -0.42) eV. **f.** Defect concentrations of BiSeI considering an annealing temperature of 550 K.

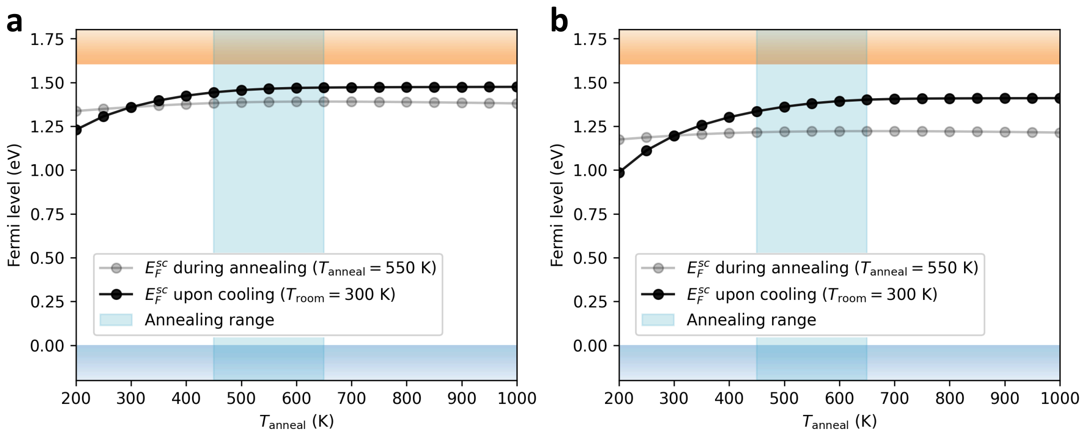

Fig. S7. Temperature dependence for BiSeI of the Fermi level during annealing synthesis conditions for **a.** Se-poor and **b.** Bi-poor conditions.

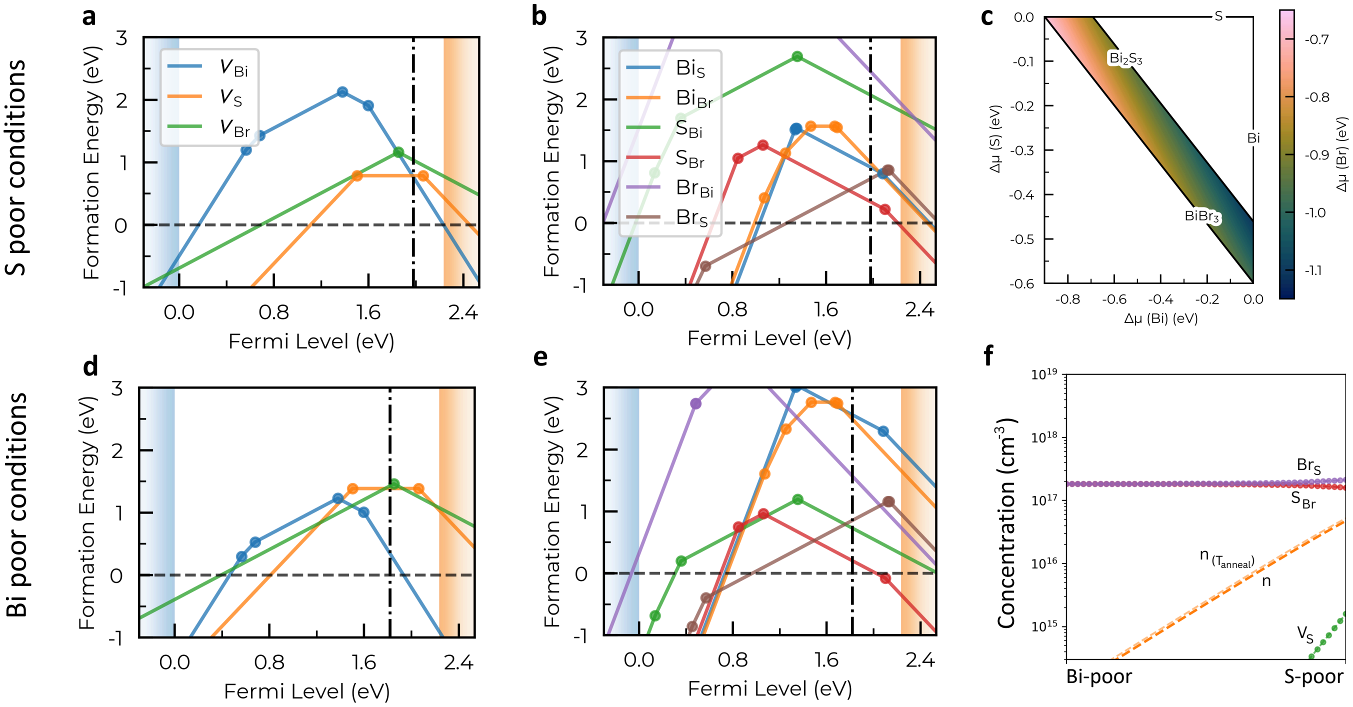

Fig. S8. Formation energies of point defects in BiSBr. **a,b.** S-poor growth conditions. **d,e.** Bi-poor growth conditions. The self-consistent Fermi level, E_F^{sc}, is represented with vertical dash-dot lines, lying at 1.98 and 1.82 eV above the VBM (blue shaded region) for S-poor and Bi-poor growth conditions, respectively (the CBM is represented by the orange shaded region). **c.** Chemical stability region, delimited by S-poor (μ_{Bi}, μ_S, μ_{Br}) = (0, -0.60, -0.97) eV and Bi-poor conditions (μ_{Bi}, μ_S, μ_{Br}) = (-0.90, 0, -0.67) eV. **f.** Defect concentrations of BiSBr considering an annealing temperature of 550 K.

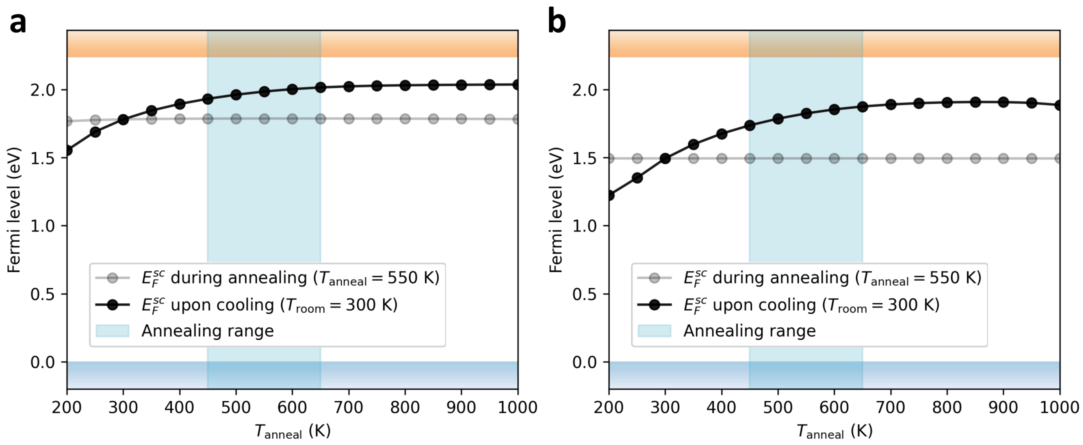

Fig. S9. Temperature dependence for BiSBr of the Fermi level during annealing synthesis conditions for **a.** S-poor and **b.** Bi-poor conditions.

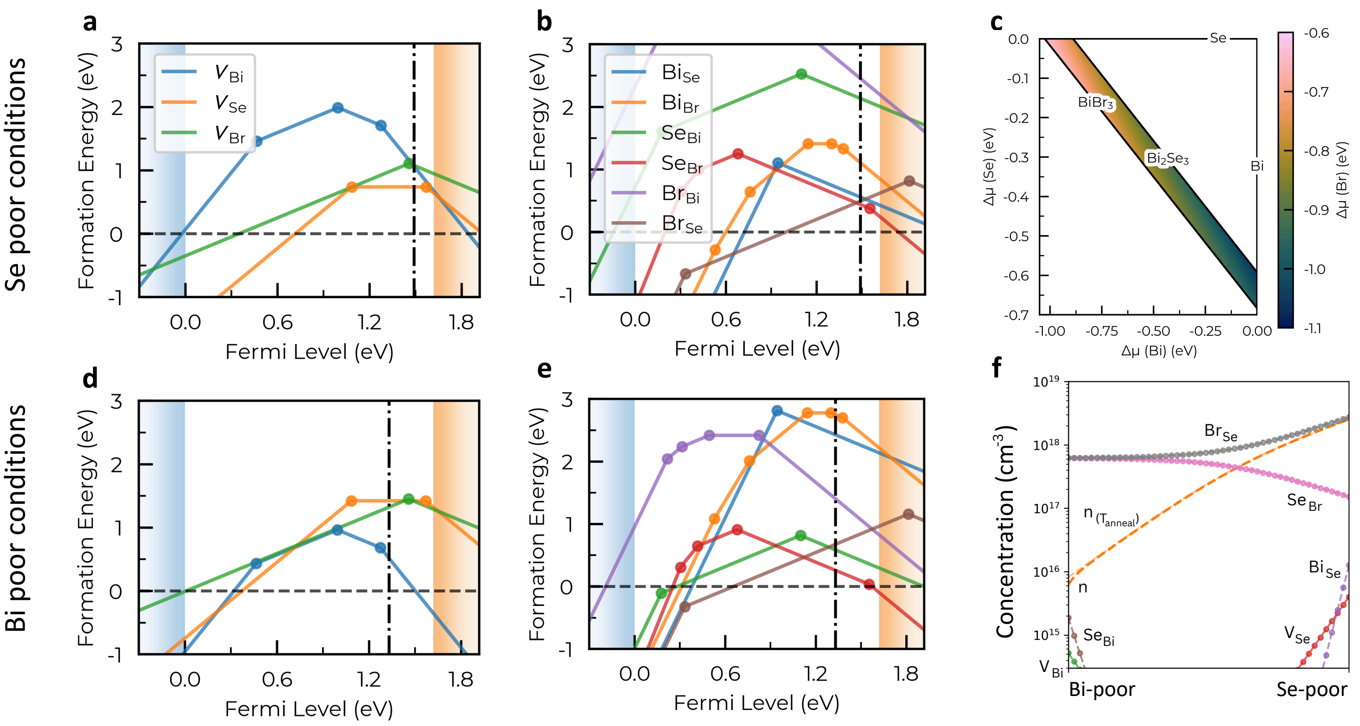

Fig. S10. Formation energies of point defects in BiSeBr. **a,b.** S-poor growth conditions. **d,e.** Bi-poor growth conditions. The self-consistent Fermi level, E_F^{sc}, is represented with vertical dash-dot lines, lying at 1.49 and 1.33 eV above the VBM (blue shaded region) for Se-poor and Bi-poor growth conditions, respectively (the CBM is represented by the orange shaded region). **c.** Chemical stability region, delimited by Se-poor (μ_{Bi}, μ_{Se}, μ_{Br}) = (0, -0.68, -0.97) eV and Bi-poor conditions (μ_{Bi}, μ_{Se}, μ_{Br}) = (-1.03, 0, -0.63) eV. **f.** Defect concentrations of BiSeBr considering an annealing temperature of 550 K.

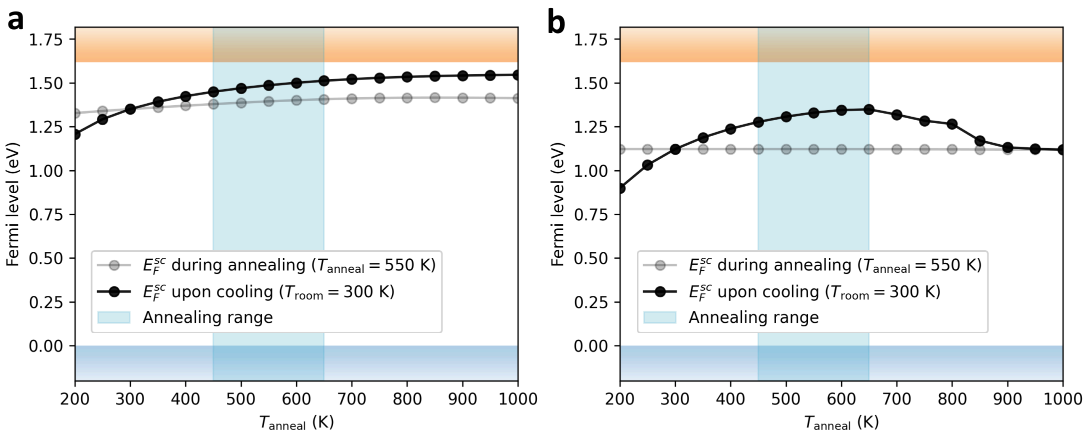

Fig. S11. Temperature dependence for BiSeBr of the Fermi level during annealing synthesis conditions for **a.** Se-poor and **b.** Bi-poor conditions.

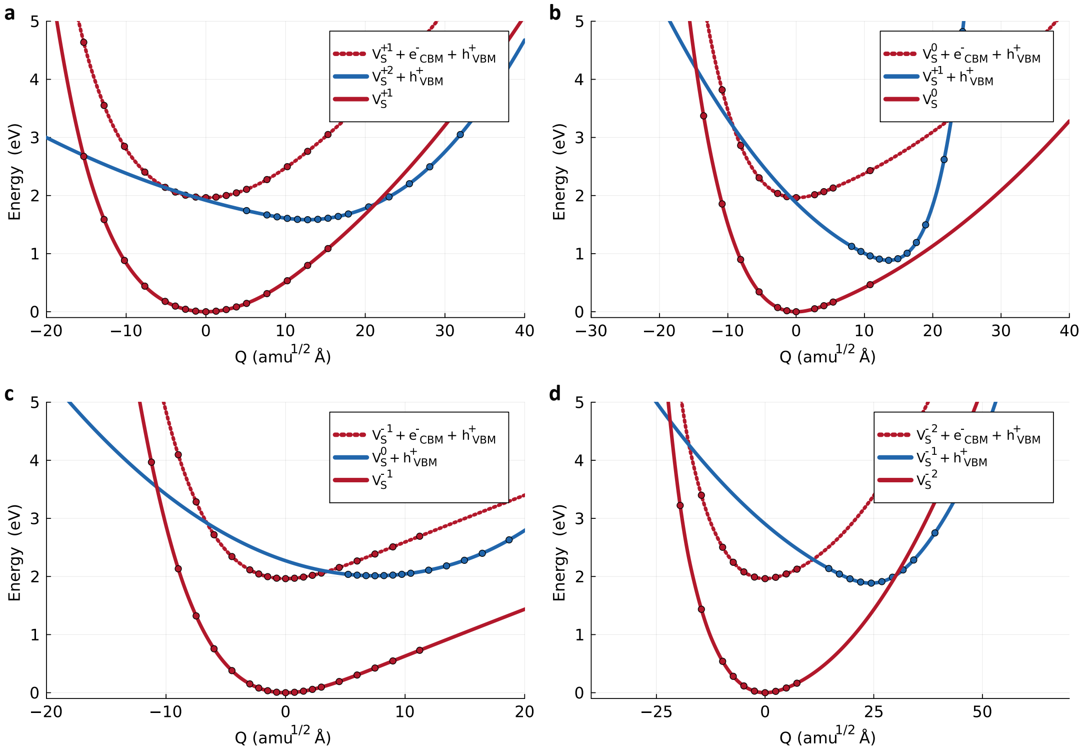

Fig. S12. Configuration coordinate diagrams for V_S in BiSI. Configuration coordinate diagrams for V_S in BiSI with transition **a.** (+1/+2), **b.** (0/+1), **c.** (-1/0) and **d.** (-2/-1). The dots represent potential energies computed from first-principles, and the solid lines are their corresponding quadratic spline interpolation and extrapolation.

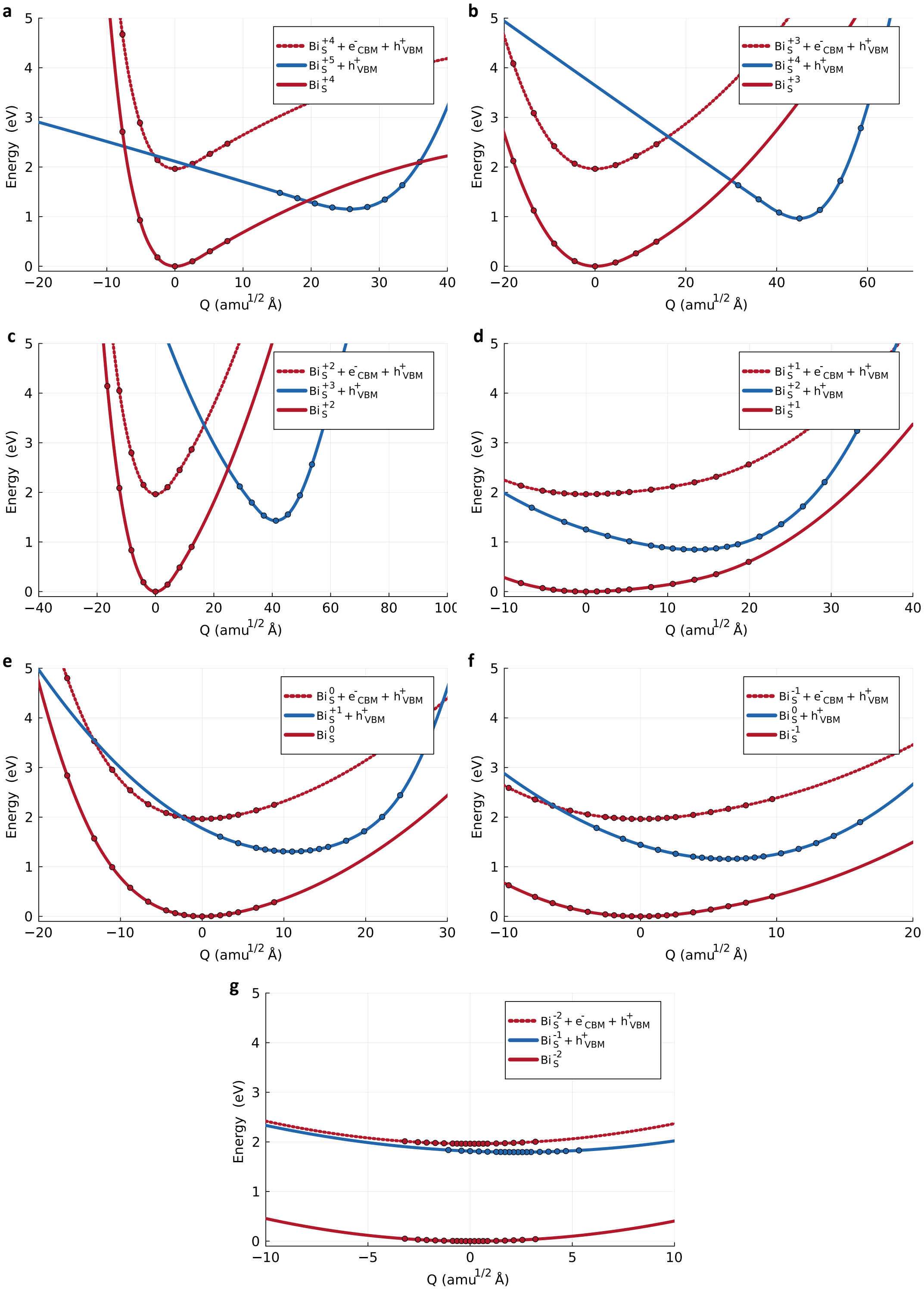

Fig. S13. Configuration coordinate diagrams for $\mathrm{Bi_S}$ in BiSI. Configuration coordinate diagrams for $\mathrm{Bi_S}$ in BiSI with transition **a.** (+4/+5), **b.** (+3/+4), **c.** (+2/+3), **d.** (+1/+2), **e.** (0/+1), **f.** (-1/0) and **g.** (-2/-1). The dots represent potential energies computed from first-principles, and the solid lines are their corresponding quadratic spline interpolation and extrapolation.

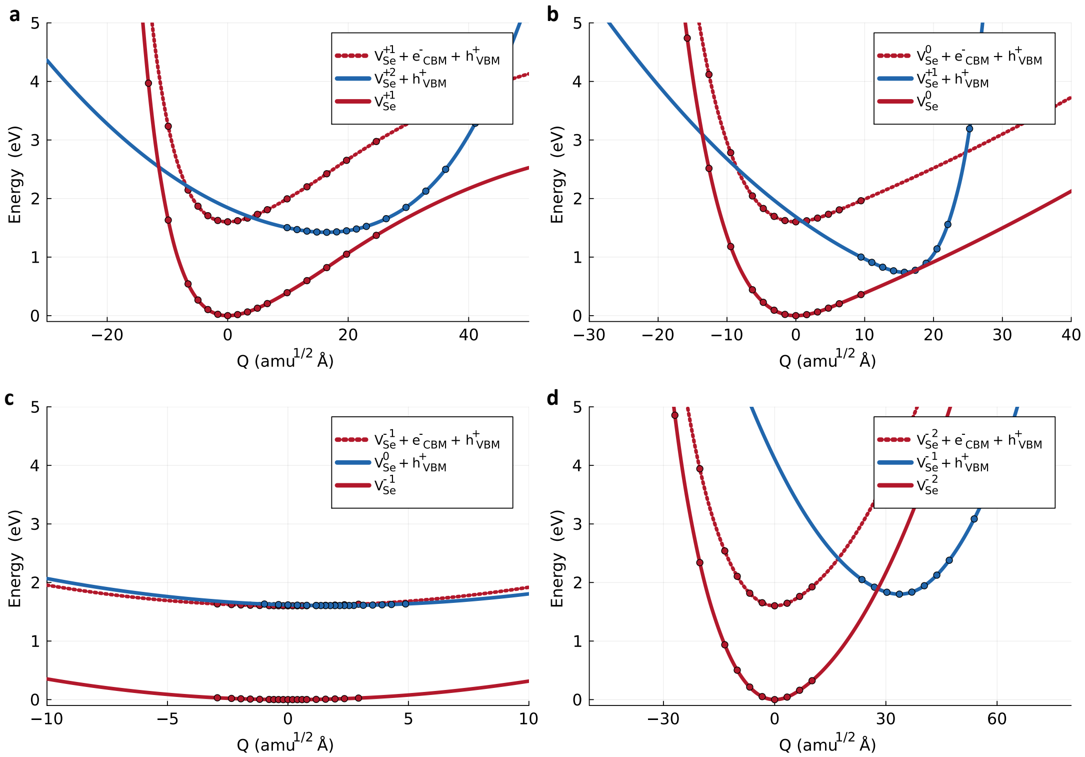

Fig. S14. Configuration coordinate diagrams for V_{Se} in BiSeI. Configuration coordinate diagrams for V_{Se} in BiSeI with transition **a.** (+1/+2), **b.** (0/+1), **c.** (-1/0) and **d.** (-2/-1). The dots represent potential energies computed from first-principles, and the solid lines are their corresponding quadratic spline interpolation and extrapolation.

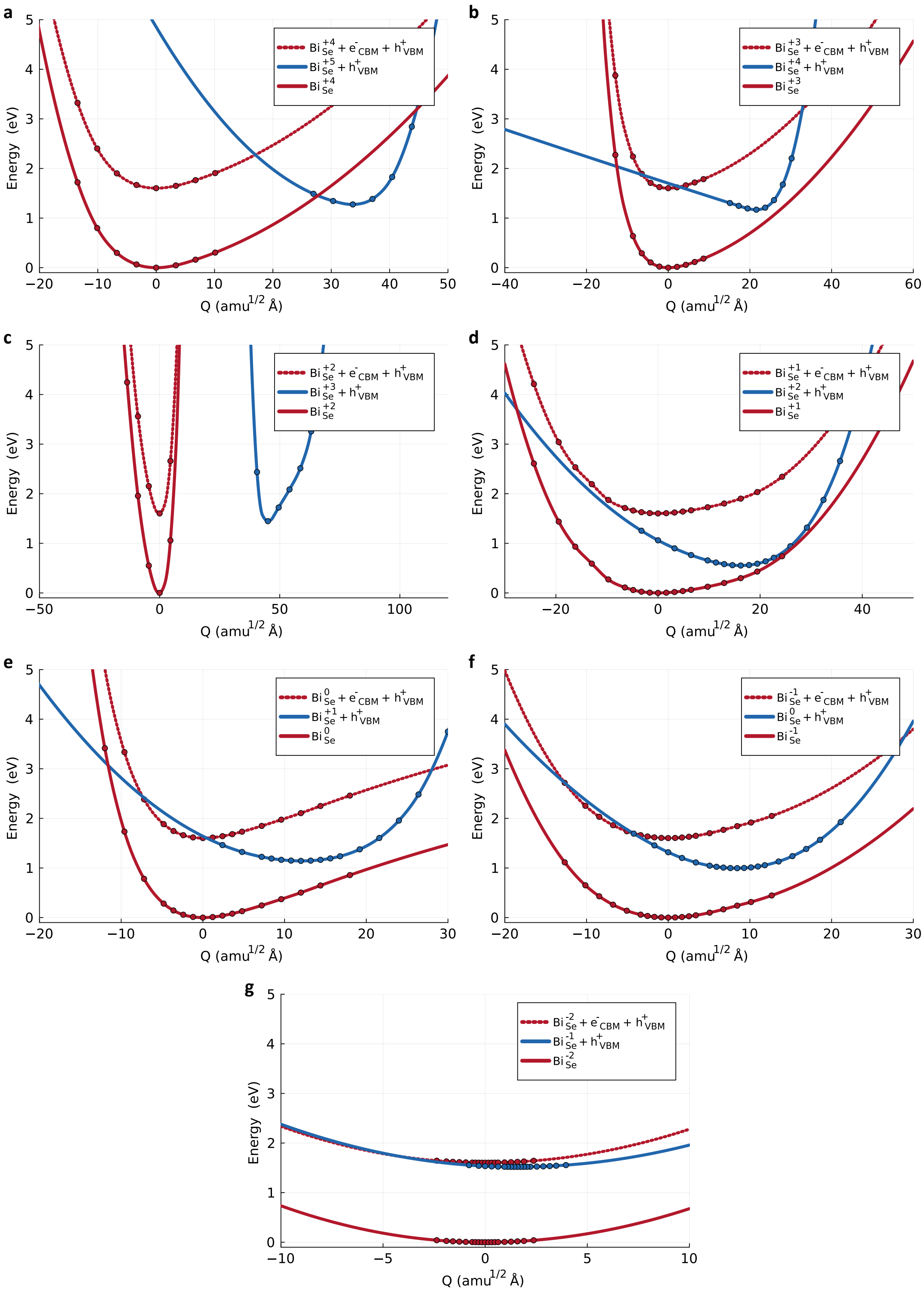

Fig. S15. Configuration coordinate diagrams for Bi_{Se} in BiSeI. Configuration coordinate diagrams for i_{Se} in BiSeI with transition **a.** (+4/+5), **b.** (+3/+4), **c.** (+2/+3), **d.** (+1/+2), **e.** (0/+1), **f.** (-1/0) and **g.** (-2/-1). The dots represent potential energies computed from first-principles, and the solid lines are their corresponding quadratic spline interpolation and extrapolation.

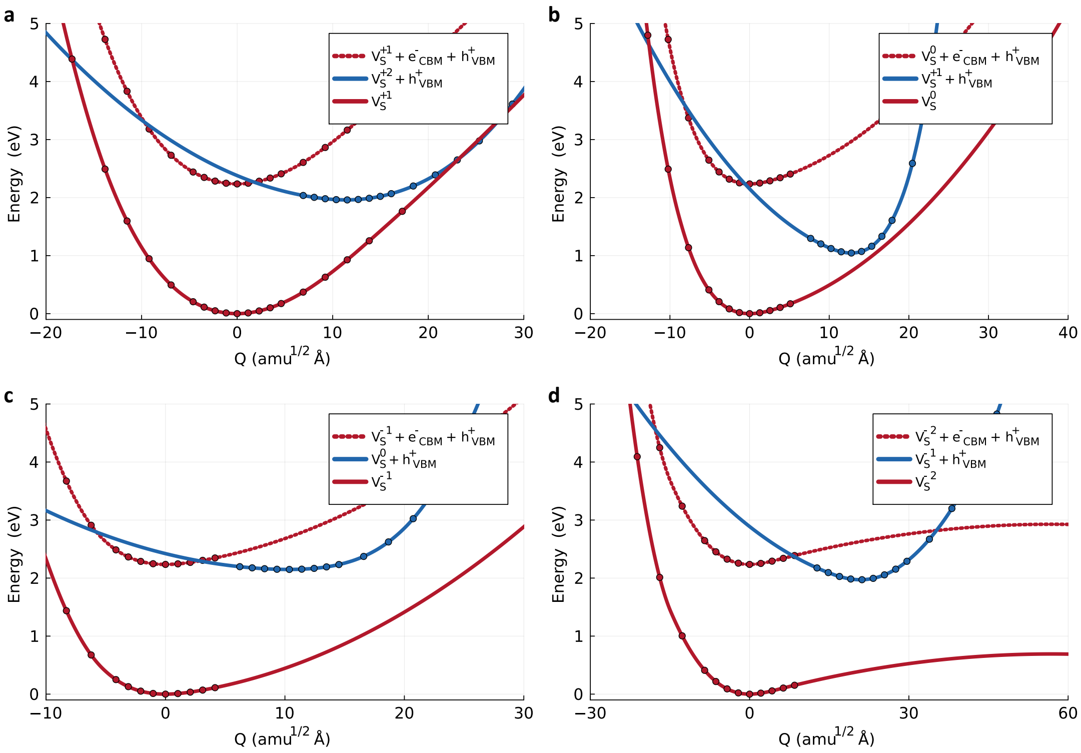

Fig. S16. Configuration coordinate diagrams for V_S in BiSBr. Configuration coordinate diagrams for V_S in BiSBr with transition **a.** (+1/+2), **b.** (0/+1), **c.** (-1/0) and **d.** (-2/-1). The dots represent potential energies computed from first-principles, and the solid lines are their corresponding quadratic spline interpolation and extrapolation.

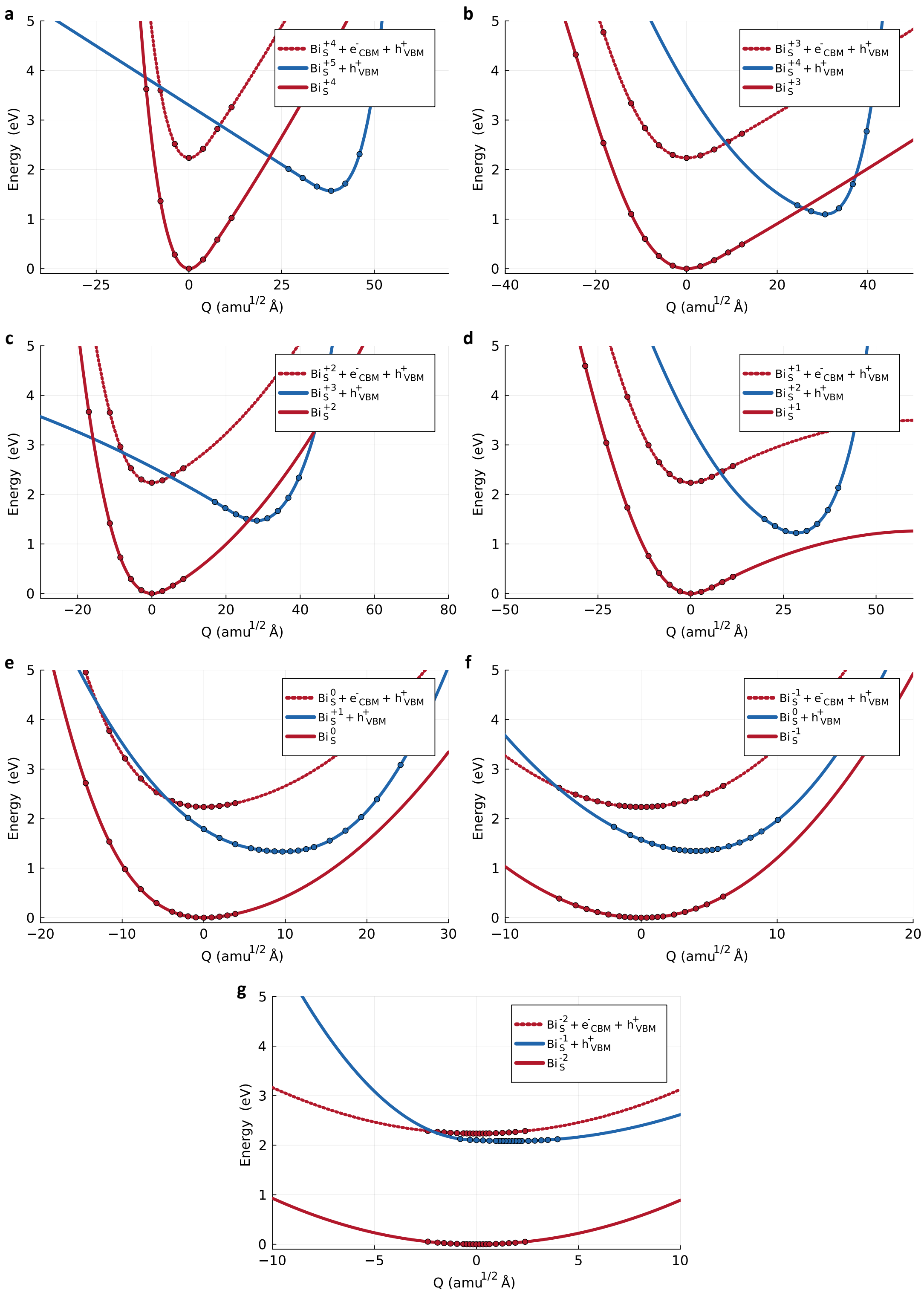

Fig. S17. Configuration coordinate diagrams for Bi_S in BiSBr. Configuration coordinate diagrams for Bi_S in BiSBr with transition **a.** (+4/+5), **b.** (+3/+4), **c.** (+2/+3), **d.** (+1/+2), **e.** (0/+1), **f.** (-1/0) and **g.** (-2/-1). The dots represent potential energies computed from first-principles, and the solid lines are their corresponding quadratic spline interpolation and extrapolation.

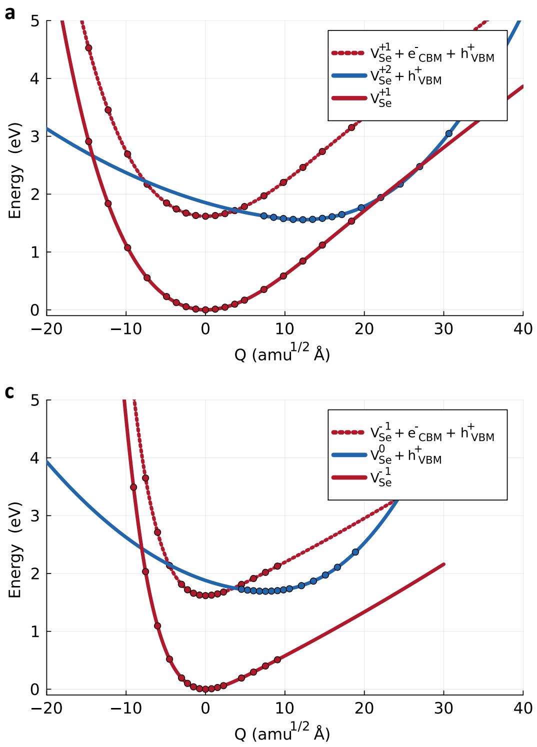

Fig. S18. Configuration coordinate diagrams for V_{Se} in BiSeBr. Configuration coordinate diagrams for V_{Se} in BiSeBr with transition **a.** (+1/+2), **b.** (0/+1), **c.** (-1/0) and **d.** (-2/-1). The dots represent potential energies computed from first-principles, and the solid lines are their corresponding quadratic spline interpolation and extrapolation.

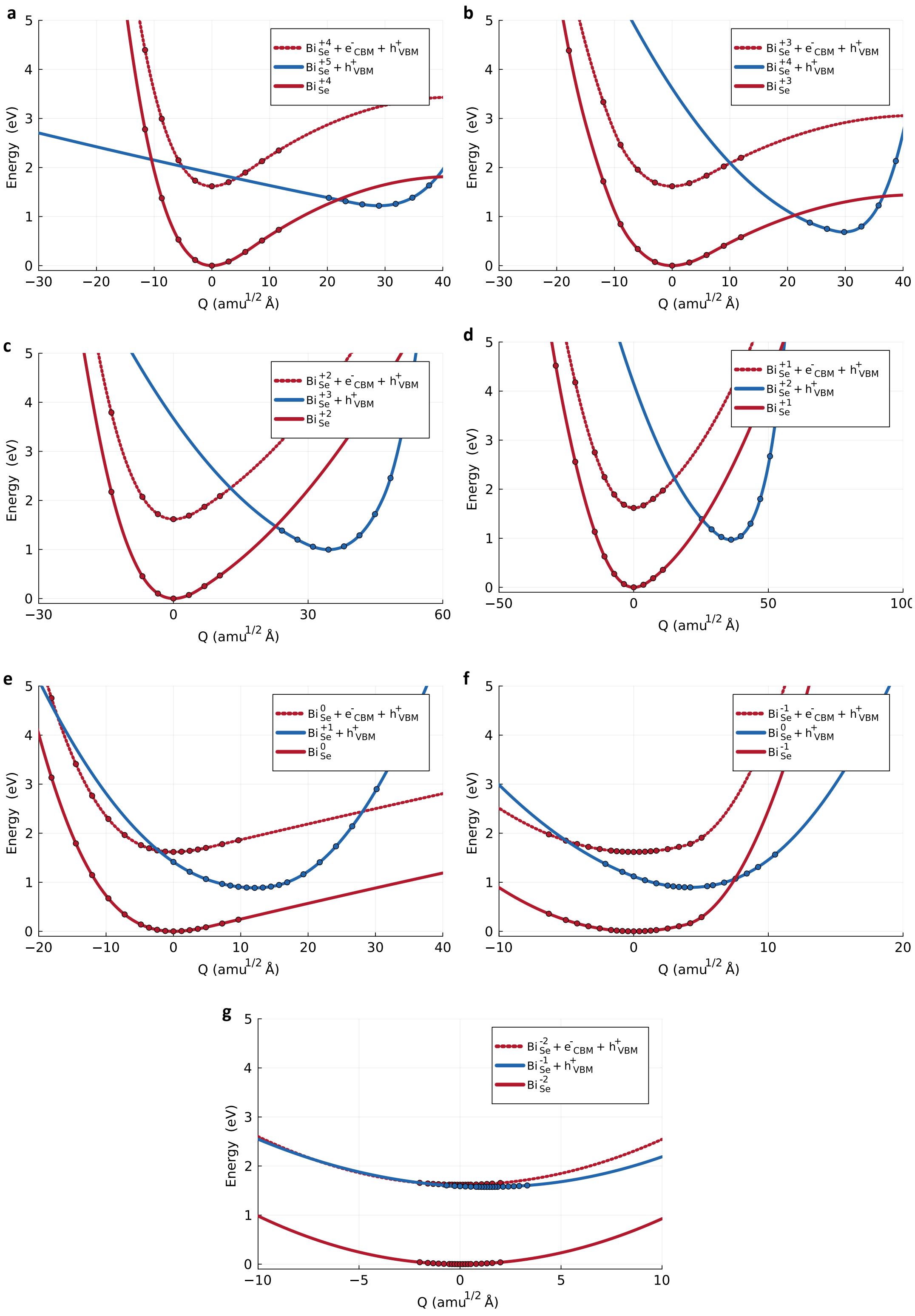

Fig. S19. Configuration coordinate diagrams for Bi_{Se} in BiSeBr. Configuration coordinate diagrams for Bi_{Se} in BiSeBr with transition **a.** (+4/+5), **b.** (+3/+4), **c.** (+2/+3), **d.** (+1/+2), **e.** (0/+1), **f.** (-1/0) and **g.** (-2/-1). The dots represent potential energies computed from first-principles, and the solid lines are their corresponding quadratic spline interpolation and extrapolation.

Material	$E_g^{\text{Exp.}}$ (eV)	E_g^{DFT} (eV)	$(a, b, c)^{\text{Exp.}}$ (Å)	$(a, b, c)^{\text{DFT}}$ (Å)
BiSI	1.45	1.49	4.17, 8.50, 10.24	4.14, 8.34, 10.02
BiSeI	1.35	1.25	4.21, 8.69, 10.56	4.22, 8.71, 10.58
BiSBr	1.84	1.83	4.06, 8.15, 9.85	4.03, 8.06, 9.52
BiSeBr	1.64	1.35	4.11, 8.19, 10.46	4.08, 8.14, 10.12

Table S1. DFT estimated [2] and experimental [3] optoelectronic and structural properties of MChX.

Defect	Charge transition	ΔQ (amu$^{1/2}$Å)	ΔE (eV)	ΔE_p (eV)	ΔE_n (eV)	$W_{if,p}$	$W_{if,n}$	C_p (cm^3/s)	C_n (cm^3/s)
V_S	-2/-1	24.42	1.89	0.14	0.33	$9.35 \cdot 10^{-3}$	$2.35 \cdot 10^{-3}$	0.00	$1.87 \cdot 10^{-12}$
V_S	-1/0	7.48	2.01	-	0.12	$5.66 \cdot 10^{-3}$	0.00	0.00	0.00
V_S	0/+1	13.54	0.89	3.30	0.01	$1.56 \cdot 10^{-2}$	$9.26 \cdot 10^{-3}$	$2.79 \cdot 10^{-13}$	$2.94 \cdot 10^{-8}$
V_S	+1/+2	12.77	1.58	0.27	0.01	$2.64 \cdot 10^{-3}$	0.00	$1.30 \cdot 10^{-24}$	0.00
Bi_S	-2/-1	2.13	1.79	11.00	-	$7.92 \cdot 10^{-4}$	0.00	0.00	0.00
Bi_S	-1/0	6.45	1.16	-	0.26	$2.57 \cdot 10^{-2}$	$7.36 \cdot 10^{-3}$	$9.63 \cdot 10^{-19}$	$2.40 \cdot 10^{-10}$
Bi_S	0/+1	11.01	1.31	3.82	0.03	$1.71 \cdot 10^{-3}$	0.00	$3.58 \cdot 10^{-21}$	0.00
Bi_S	+1/+2	13.27	0.85	10.70	2.95	$9.57 \cdot 10^{-3}$	$7.00 \cdot 10^{-3}$	$5.49 \cdot 10^{-18}$	$3.44 \cdot 10^{-22}$
Bi_S	+2/+3	41.24	1.43	1.05	1.39	$1.47 \cdot 10^{-2}$	0.00	$1.23 \cdot 10^{-33}$	0.00
Bi_S	+3/+4	45.02	0.96	0.76	0.65	$2.76 \cdot 10^{-3}$	$7.96 \cdot 10^{-4}$	$2.15 \cdot 10^{-35}$	$2.60 \cdot 10^{-36}$
Bi_S	+4/+5	25.68	1.15	0.16	0.07	$6.27 \cdot 10^{-3}$	0.00	$1.06 \cdot 10^{-8}$	0.00

Table S2. Calculated BiSI capture coefficients for each charge-carrier process involving V_S and Bi_S, being ΔE the transition energy above the VBM, and key parameters used to calculate the carrier capture coefficients in each transition. $\Delta E_{n/p}$ and $W_{if,n/p}$ are the energy barriers and electron-phonon coupling matrix elements for electron and hole capture processes, respectively.

Defect	Charge transition	ΔQ (amu$^{1/2}$Å)	ΔE (eV)	ΔE_p (eV)	ΔE_n (eV)	$W_{if,p}$	$W_{if,n}$	C_p (cm^3/s)	C_n (cm^3/s)
V_{Se}	-2/-1	33.64	0.61	0.09	0.81	$1.10 \cdot 10^{-3}$	$6.88 \cdot 10^{-4}$	0.00	$8.90 \cdot 10^{-21}$
V_{Se}	-1/0	1.95	0.00	11.90	0.00	$9.33 \cdot 10^{-4}$	$2.45 \cdot 10^{-4}$	$2.80 \cdot 10^{-20}$	$6.71 \cdot 10^{-9}$
V_{Se}	0/+1	15.77	0.87	2.36	0.01	$5.32 \cdot 10^{-4}$	$6.21 \cdot 10^{-4}$	$5.89 \cdot 10^{-8}$	$1.91 \cdot 10^{-10}$
V_{Se}	+1/+2	16.44	0.26	1.12	0.08	$1.25 \cdot 10^{-3}$	$5.41 \cdot 10^{-4}$	$2.98 \cdot 10^{-20}$	$1.24 \cdot 10^{-9}$
Bi_{Se}	-2/-1	1.58	0.13	7.93	0.13	$3.63 \cdot 10^{-4}$	$9.93 \cdot 10^{-4}$	$1.15 \cdot 10^{-23}$	$5.53 \cdot 10^{-10}$
Bi_{Se}	-1/0	8.45	0.70	3.30	0.10	$6.87 \cdot 10^{-4}$	$3.71 \cdot 10^{-4}$	$8.83 \cdot 10^{-21}$	$5.88 \cdot 10^{-10}$
Bi_{Se}	0/+1	12.00	0.46	1.93	0.00	$5.76 \cdot 10^{-4}$	$7.31 \cdot 10^{-4}$	$4.21 \cdot 10^{-20}$	$7.47 \cdot 10^{-11}$
Bi_{Se}	+1/+2	16.20	3.83	3.15	2.78	$6.68 \cdot 10^{-4}$	$6.36 \cdot 10^{-4}$	$1.34 \cdot 10^{-16}$	$3.09 \cdot 10^{-23}$
Bi_{Se}	+2/+3	45.05	69.10	68.50	68.90	$4.77 \cdot 10^{-4}$	$1.88 \cdot 10^{-4}$	$5.64 \cdot 10^{-38}$	$4.63 \cdot 10^{-38}$
Bi_{Se}	+3/+4	21.58	14.41	0.00	1.17	$1.74 \cdot 10^{-3}$	$8.67 \cdot 10^{-5}$	0.00	$1.38 \cdot 10^{-11}$
Bi_{Se}	+4/+5	33.69	1.00	0.18	0.67	$8.25 \cdot 10^{-4}$	$1.46 \cdot 10^{-3}$	$1.48 \cdot 10^{-43}$	$4.12 \cdot 10^{-19}$

Table S3. Calculated BiSeI capture coefficients for each charge-carrier process involving V_{Se} and Bi_{Se}, being ΔE the transition energy above the VBM, and key parameters used to calculate the carrier capture coefficients in each transition. $\Delta E_{n/p}$ and $W_{if,n/p}$ are the energy barriers and electron-phonon coupling matrix elements for electron and hole capture processes, respectively.

Defect	Charge transition	ΔQ (amu$^{1/2}$Å)	ΔE (eV)	ΔE_p (eV)	ΔE_n (eV)	$W_{if,p}$	$W_{if,n}$	C_p (cm^3/s)	C_n (cm^3/s)
V_S	-2/-1	21.17	1.97	3.20	0.15	$1.87 \cdot 10^{-2}$	$5.89 \cdot 10^{-3}$	0.00	$4.66 \cdot 10^{-9}$
V_S	-1/0	10.37	2.15	-	0.06	$3.78 \cdot 10^{-3}$	0.00	0.00	0.00
V_S	0/+1	12.78	1.05	3.58	0.00	$2.82 \cdot 10^{-2}$	$3.70 \cdot 10^{-4}$	$4.71 \cdot 10^{-13}$	$2.20 \cdot 10^{-11}$
V_S	+1/+2	11.52	1.96	0.71	0.03	$3.09 \cdot 10^{-3}$	0.00	0.00	0.00
Bi_S	-2/-1	1.59	2.08	8.11	0.04	$1.47 \cdot 10^{-3}$	0.00	$6.79 \cdot 10^{-23}$	0.00
Bi_S	-1/0	4.02	1.35	-	0.41	$2.50 \cdot 10^{-2}$	$4.72 \cdot 10^{-3}$	$4.58 \cdot 10^{-20}$	$1.36 \cdot 10^{-13}$
Bi_S	0/+1	9.65	1.34	5.75	0.18	$6.24 \cdot 10^{-3}$	0.00	$1.95 \cdot 10^{-19}$	0.00
Bi_S	+1/+2	28.44	1.22	-	0.21	$2.86 \cdot 10^{-2}$	$1.14 \cdot 10^{-2}$	0.00	$1.69 \cdot 10^{-9}$
Bi_S	+2/+3	28.32	1.47	0.02	0.13	$1.44 \cdot 10^{-2}$	0.00	$1.79 \cdot 10^{-51}$	0.00
Bi_S	+3/+4	30.57	1.10	0.13	0.30	$1.25 \cdot 10^{-3}$	$9.30 \cdot 10^{-4}$	$5.99 \cdot 10^{-10}$	$1.80 \cdot 10^{-13}$
Bi_S	+4/+5	38.34	1.57	0.69	0.66	$1.00 \cdot 10^{-2}$	$1.23 \cdot 10^{-6}$	$2.54 \cdot 10^{-33}$	$3.66 \cdot 10^{-30}$

Table S4. Calculated BiSBr capture coefficients for each charge-carrier process involving V_S and Bi_S, being ΔE the transition energy above the VBM, and key parameters used to calculate the carrier capture coefficients in each transition. $\Delta E_{n/p}$ and $W_{if,n/p}$ are the energy barriers and electron-phonon coupling matrix elements for electron and hole capture processes, respectively.

Defect	Charge transition	ΔQ (amu$^{1/2}$Å)	ΔE (eV)	ΔE_p (eV)	ΔE_n (eV)	$W_{if,p}$	$W_{if,n}$	C_p (cm^3/s)	C_n (cm^3/s)
V_{Se}	-2/-1	21.44	1.44	0.29	0.19	$9.91 \cdot 10^{-3}$	$4.14 \cdot 10^{-3}$	0.00	$1.31 \cdot 10^{-9}$
V_{Se}	-1/0	7.55	1.69	-	0.13	$2.15 \cdot 10^{-3}$	0.00	$4.09 \cdot 10^{-22}$	0.00
V_{Se}	0/+1	14.98	0.61	0.01	0.00	$1.70 \cdot 10^{-2}$	$5.21 \cdot 10^{-4}$	$2.95 \cdot 10^{-5}$	$2.64 \cdot 10^{-11}$
V_{Se}	+1/+2	12.26	1.56	0.38	0.10	$1.89 \cdot 10^{-3}$	0.00	$3.82 \cdot 10^{-18}$	0.00
Bi_{Se}	-2/-1	1.33	1.57	7.44	0.03	$2.64 \cdot 10^{-3}$	0.00	$5.82 \cdot 10^{-23}$	0.00
Bi_{Se}	-1/0	4.20	0.90	0.17	0.23	$3.27 \cdot 10^{-2}$	$4.35 \cdot 10^{-3}$	$8.85 \cdot 10^{-9}$	$6.50 \cdot 10^{-11}$
Bi_{Se}	0/+1	12.07	0.89	-	0.03	$7.80 \cdot 10^{-3}$	0.00	$1.23 \cdot 10^{-17}$	0.00
Bi_{Se}	+1/+2	36.15	0.97	0.40	0.60	$4.94 \cdot 10^{-3}$	$3.21 \cdot 10^{-3}$	$1.85 \cdot 10^{-34}$	$9.83 \cdot 10^{-17}$
Bi_{Se}	+2/+3	34.54	1.00	0.47	0.63	$8.08 \cdot 10^{-3}$	0.00	$3.57 \cdot 10^{-15}$	0.00
Bi_{Se}	+3/+4	29.81	0.69	0.34	0.47	$7.88 \cdot 10^{-3}$	$2.91 \cdot 10^{-3}$	$1.37 \cdot 10^{-12}$	$6.55 \cdot 10^{-15}$
Bi_{Se}	+4/+5	28.95	1.22	0.12	0.16	$5.23 \cdot 10^{-3}$	$4.60 \cdot 10^{-7}$	$1.55 \cdot 10^{-36}$	$5.52 \cdot 10^{-18}$

Table S5. Calculated BiSeBr capture coefficients for each charge-carrier process involving V_{Se} and Bi_{Se}, being ΔE the transition energy above the VBM, and key parameters used to calculate the carrier capture coefficients in each transition. $\Delta E_{n/p}$ and $W_{if,n/p}$ are the energy barriers and electron-phonon coupling matrix elements for electron and hole capture processes, respectively.

**Material**	**Limit**	**Defect**	V_{oc} (V)	FF (%)	η (%)
BiSI	Bi-poor	V_S	1.66	92.08	23.66
		Bi_S	1.66	92.08	23.66
	S-poor	V_S	1.66	90.66	23.20
		Bi_S	1.66	92.08	23.63
BiSeI	Bi-poor	V_{Se}	1.33	90.54	30.39
		Bi_{Se}	1.33	90.54	30.39
	Se-poor	V_{Se}	1.08	88.81	24.23
		Bi_{Se}	1.33	90.54	30.39
BiSBr	Bi-poor	V_S	1.92	92.94	17.65
		Bi_S	1.92	92.94	17.65
	S-poor	V_S	1.91	91.25	17.26
		Bi_S	1.92	92.94	17.65
BiSeBr	Bi-poor	V_{Se}	1.35	90.66	27.96
		Bi_{Se}	1.35	90.66	27.96
	Se-poor	V_{Se}	0.91	87.23	18.21
		Bi_{Se}	1.14	89.24	23.17

Table S6. Separate contribution of chalcogen vacancies and Bi_{Ch} antisites to the photovoltaic performance of MChX in the trap-mediated limit.

**Material**	**Functional**	E_g (eV)	V_{oc} (V)	FF (%)	η (%)
BiSI	$\mathrm{PBE_{sol}}$+D3	1.49	1.23	89.93	31.51
	HSE+D3+SOC	1.96	1.66	92.08	23.63
BiSeI	$\mathrm{PBE_{sol}}$+D3	1.25	1.00	88.20	32.54
	HSE+D3+SOC	1.60	1.33	90.54	30.39
BiSBr	$\mathrm{PBE_{sol}}$+D3	1.83	1.55	91.61	24.63
	HSE+D3+SOC	2.24	1.92	92.94	17.65
BiSeBr	$\mathrm{PBE_{sol}}$+D3	1.35	1.10	89.08	30.09
	HSE+D3+SOC	1.62	1.35	90.66	27.96

Table S7. Photovoltaic performance of MChX (at room temperature for a 700 nm absorber layer) in the radiative limit, considering bandgaps which come from $\mathrm{PBE_{sol}}$+D3 (top) and HSE+D3+SOC (bottom) geometrical optimizations. $\mathrm{PBE_{sol}}$+D3 bandgaps are computed using $\mathrm{HSE_{sol}}$+SOC on top of the relaxed geometries.

**Supercell**	$E_T(\mathrm{V}_{\mathrm{Se}}^{-2}) - E_0$	$E_T(\mathrm{V}_{\mathrm{Se}}^{-1}) - E_0)$	$E_T(\mathrm{V}_{\mathrm{Se}}^{0}) - E_0$	$E_T(\mathrm{V}_{\mathrm{Se}}^{+1}) - E_0$	$E_T(\mathrm{V}_{\mathrm{Se}}^{+2}) - E_0$
$2 \times 1 \times 1$	3.23	3.40	3.60	3.73	3.93
$3 \times 2 \times 1$	3.59	3.66	3.73	3.79	3.86
$5 \times 3 \times 2$	3.77	3.78	3.80	3.81	3.82

Table S8. Size effects test on the formation energy of V_{Se} for BiSeBr. E_0 and $E_T(\mathrm{V}_{\mathrm{Se}}^{\mathrm{q}})$ represent the energy (in eV) of the pristine and defected systems, respectively, and q indicates the charge state of the Se vacancy. Calculations were performed with the GGA-PBE exchange-correlation functional.

SUPPLEMENTARY DISCUSSION

The conducted DFT geometry optimizations resulted in crystal symmetries and lattice parameters that are in excellent agreement with the experimental results (Supplementary Table I). The theoretical lattice parameters were estimated at zero-temperature conditions, whilst the experimental values have been obtained at room temperature. Therefore, the systematic $\approx 3\%$ underestimation of the experimental lattice parameters is likely originated by thermal lattice expansion effects.

Electron effective masses [3] typically lie between 0.3 and 3.3, being 0.34 for BiSeBr along the k-path $\Lambda \rightarrow X$ the smallest mass and 3.25 for SbSeI along $\Lambda \rightarrow T$ the largest one. Hole masses, on the other hand, are found to remain between 0.2 and 2.2, presenting SbSI the smallest mass with 0.24 for the k-path $\Lambda \rightarrow \Gamma$, and the largest one for BiSBr with 2.14 in $\Lambda \rightarrow \Gamma$. It is noted that effective masses from Br-containing elements, in comparison to I-based chalcohalides, are larger for holes and smaller for electrons.

The conduction and valence bands are depicted in yellow and blue, respectively, with the orbital contributions shown in the accompanying density of states diagrams (the Fermi level is set to zero, Supplementary Fig. 2). The top of the valence band (VBM) and the bottom of the conduction band (CBM) are highlighted by green and red dots, respectively. All four compounds are found to exhibit predominantly indirect bandgaps, meaning that the VBM and CBM occur at different points in reciprocal space [3]. Nevertheless, the minimum direct bandgaps are very close in energy to the indirect ones, differing by less than 0.2 eV. This small separation leads to quasi-direct bandgap behavior, which is known to enhance radiative recombination by mitigating momentum-mismatch constraints. The electronic density of states further reveals that p-orbitals from halogen and chalcogen atoms dominate the VBM, while p-orbitals from pnictogen atoms primarily contribute to the CBM [2].

All defective atomic structures in this work were fully optimized at the HSE+D3+SOC level, which yields more reliable defect energetics and geometries [4, 5]. This choice, however, systematically increases the bandgap of MChX by about 0.4 eV compared to previous $\mathrm{HSE_{sol}}$+SOC calculations performed on $\mathrm{PBE_{sol}}$+D3-relaxed structures [2], which are in closer agreement with experiments [3] and may therefore be considered as reference. Consequently, the calculated radiative-limit efficiencies are spuriously reduced for all materials (Supplementary Table VII): the drop is modest for BiSeI and BiSeBr ($\sim$2%), but more pronounced for BiSI and BiSBr ($\sim$8%). Other photovoltaic parameters are only marginally affected, with FF increasing by about 3% and V_{oc} by ≈ 0.4 V.

SUPPLEMENTARY REFERENCES

[1] A. M. Ganose, A. J. Jackson, and D. O. Scanlon, Journal of Open Source Software **3**, 717 (2018).
[2] C. López, I. Caño, D. Rovira, P. Benítez, J. M. Asensi, Z. Jehl, J.-L. Tamarit, E. Saucedo, and C. Cazorla, Adv. Funct. Mater. **34**, 2406678 (2024).
[3] I. Caño, A. Navarro-Güell, E. Maggi, A. Gon Medaille, D. Rovira, A. Jimenez-Arguijo, O. Segura, A. Torrens, M. Jimenez, C. López, P. Benítez, C. Cazorla, Z. Jehl, Y. Gong, J.-M. Asensi, L. Calvo-Barrio, L. Soler, J. Llorca, J.-L. Tamarit, B. Galiana, M. Dimitrievska, N. Ruiz-Marín, H. Z. Chun, L. Wong, J. Puigdollers, M. Placidi, and E. Saucedo, Small **21**, e05430 (2025).
[4] J. Pan, W. K. Metzger, and S. Lany, Phys. Rev. B **98**, 054108 (2018).
[5] C. López, S. R. Kavanagh, P. Benítez, E. Saucedo, A. Walsh, D. O. Scanlon, and C. Cazorla, ACS Energy Lett. **10**, 3562 (2025).
